# Wrestling and Wrapping: A Perspective on SUMO Proteins in Schwann Cells

**DOI:** 10.3390/biom11071055

**Published:** 2021-07-19

**Authors:** Iman F. Fergani, Luciana R. Frick

**Affiliations:** 1 Hunter James Kelly Research Institute, Jacobs School of Medicine and Biomedical Sciences, University at Buffalo, State University of New York, Buffalo, NY 14203, USA; imanfara@buffalo.edu; 2 Department of Neurology, Jacobs School of Medicine and Biomedical Sciences, University at Buffalo, State University of New York, Buffalo, NY 14203, USA

**Keywords:** Schwann cells, myelin, SUMO proteins, SUMOylation, post-translational modifications, development, peripheral neuropathies, nerve injury

## Abstract

Schwann cell development and peripheral nerve myelination are finely orchestrated multistep processes; some of the underlying mechanisms are well described and others remain unknown. Many posttranslational modifications (PTMs) like phosphorylation and ubiquitination have been reported to play a role during the normal development of the peripheral nervous system (PNS) and in demyelinating neuropathies. However, a relatively novel PTM, SUMOylation, has not been studied in these contexts. SUMOylation involves the covalent attachment of one or more small ubiquitin-like modifier (SUMO) proteins to a substrate, which affects the function, cellular localization, and further PTMs of the conjugated protein. SUMOylation also regulates other proteins indirectly by facilitating non-covalent protein–protein interaction via SUMO interaction motifs (SIM). This pathway has important consequences on diverse cellular processes, and dysregulation of this pathway has been reported in several diseases including neurological and degenerative conditions. In this article, we revise the scarce literature on SUMOylation in Schwann cells and the PNS, we propose putative substrate proteins, and we speculate on potential mechanisms underlying the possible involvement of this PTM in peripheral myelination and neuropathies.

## 1. Introduction

### 1.1. Mechanisms of Schwann Cell Development and Myelination in the Peripheral Nervous System

During the development of the peripheral nervous system (PNS), Schwann cell precursors originate from the neural crest and start migrating along axons to reach their target tissue. Immature Schwann cells choose larger axons to myelinate in a process called radial sorting; pro-myelinating Schwann cells establish a 1:1 relationship with axons and subsequently deposit the myelin sheath. During the process of myelination, myelinating Schwann cells interact with both the axon and the extracellular matrix in a complex and finely orchestrated manner, and the molecular mechanisms involved have been extensively reviewed in [1].

Among the mechanisms underlying Schwann cell myelination, different post-translational modifications (PTMs) of proteins play crucial roles, not only in normal development but also in pathological conditions. There are many examples of phosphorylation by kinases involved in myelination in health and disease, e.g., [2,3,4]. Other reports describe important physiological and pathological roles for ubiquitination: it is involved in the maintenance of Schwann cell homeostasis, and it is dysregulated in demyelinating peripheral neuropathies [5]. A recent paper also showed that Schwann cell O-GlcNAc glycosylation is required for myelin maintenance and axonal integrity [6]. However, whether SUMOylation, another important PTM, is involved in the normal formation of myelin and/or in the pathophysiology of peripheral neuropathies is unknown.

### 1.2. SUMOylation Machinery, Mechanisms, and Functions

**Figure 1:**
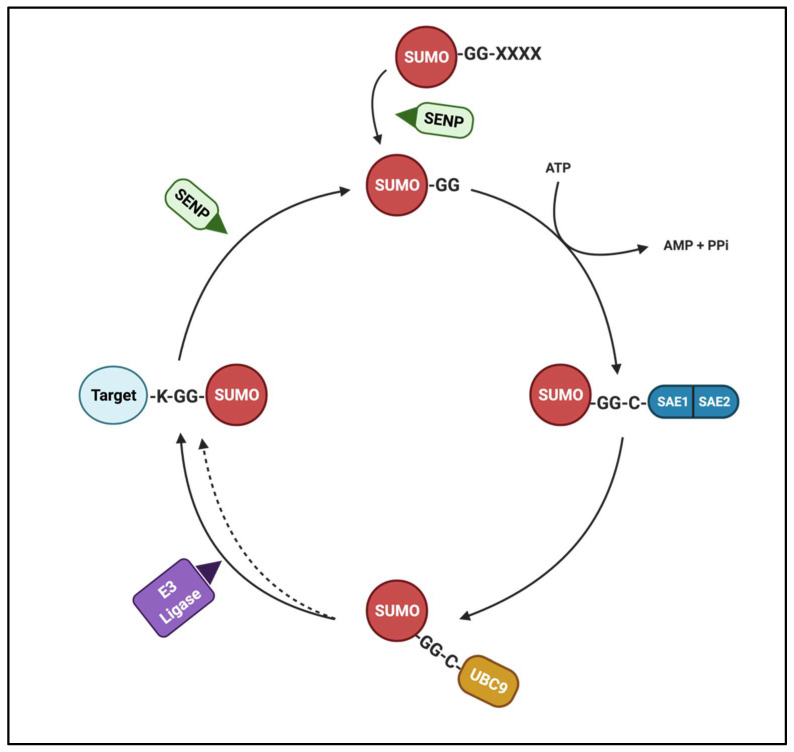
The biochemistry of the SUMOylation pathway. SUMO precursor proteins undergo a maturation process mediated by SENPs that exposes the GG motif. Next, ATP dependent activation of SAE1/SAE2 (E1 SUMO activating enzyme) transfers the mature SUMO to *Ubc9* (E2 SUMO conjugating enzyme). *Ubc9* transfers SUMO to an acceptor lysine moiety in the target protein with (and sometimes without) the involvement of a E3 SUMO ligase enzyme. DeSUMOylation of SUMO-conjugated proteins is facilitated by SENPs.

SUMOylation is a biological process in which proteins are post-translationally modified by the covalent attachment of a SUMO (Small Ubiquitin-like MOdifier) protein or a chain thereof. In rodents, this family has three members: *Sumo1*, *Sumo2*, and *Sumo3*, whereas in humans *Sumo4* has also been identified. SUMOylation is directed by an enzymatic cascade analogous to that involved in ubiquitination [7]. SUMO precursors undergo a maturation process that exposes a free C-terminal di-glycine (GG) motif. The SUMO protein is then covalently attached to an acceptor lysine residue in the substrate protein through a series of intermediate steps, as illustrated in Figure 1. *Ubc9* is the only enzyme in the conjugation step, thus it is a convenient target for genetic manipulations. SUMO conjugation usually occurs in a consensus motif ψ-K-X-E (where ψ is a hydrophobic amino acid, and X is any amino acid residue), but attachment of SUMO proteins to lysine residues outside consensus sites has been reported [8,9]. The determinants for the specificity of *Sumo1*, *2*, or *3* conjugation remain unknown, but it is suggested to be dictated by SUMO ligases. Mutation of the GG motif in the SUMO protein or the acceptor lysine in the substrate abrogates SUMOylation and has been widely used as a research strategy, as discussed below. Similar to other PTMs, SUMOylation is a dynamic and reversible process with deSUMOylation performed by SUMO-specific proteases (SENPs, Figure 1). These enzymes are also involved in the maturation of precursors and the edition of *Sumo2/3* chains. The family of SENPs comprises at least six known members with redundant and pleiotropic functions [10]. 

Unlike ubiquitination, SUMO conjugation does not tag proteins for degradation. Instead, SUMOylation has many functions, including control of protein stability, protein-protein interaction, protein localization and transport to subcellular compartments, thus regulating cellular processes such as transcription, proliferation, apoptosis and response to stress [11]. This is not only achieved by covalent attachment but also through SUMO-interaction motifs (SIMs) that facilitate non-covalent interactions between proteins. *Sumo2* and *Sumo3* show a high degree of similarity to each other (96% identical in amino acid sequence), and are distinct from *Sumo1* in homology, relative expression levels and subcellular localization [12]. For these reasons, *Sumo2* and *Sumo3* have been proposed to be more functionally similar [13]. All components of the SUMOylation machinery are differentially regulated in the developing brain [14]. The expression of SUMO proteins in the PNS has not been reported yet. Importantly, *Ubc9* constitutive knockout mice are embryonically lethal [15] and *Sumo2* knockout embryos also die in utero. On the contrary, *Sumo1* and *Sumo3* knockout mice are viable and fertile [16,17]. This suggests that *Sumo2* may be the most important SUMO protein in development. 

**Figure 2:**
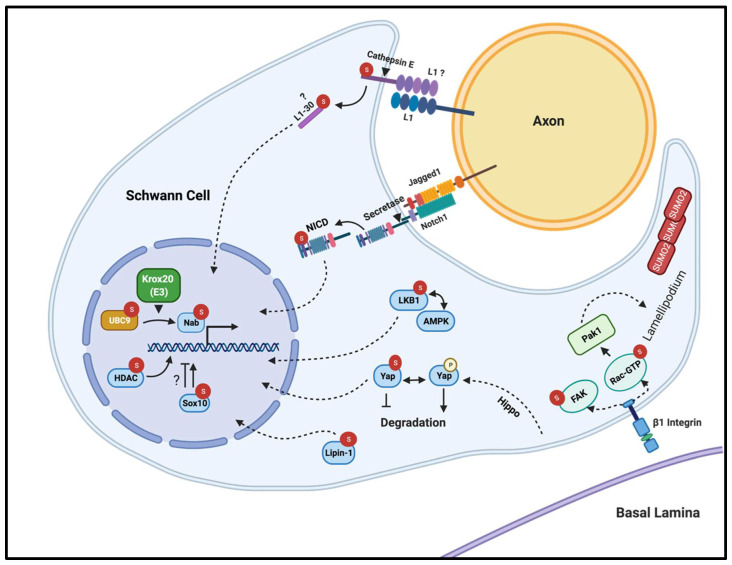
Putative substrates and speculated consequences of their SUMOylation in Schwann cells during PNS development. *Sumo2* was found to be enriched in Schwann cell protrusions extending towards neuronal preparations, suggesting that SUMOylation may be involved in axoglial interaction and myelination. Rac1 is necessary for proper radial sorting and myelination by Schwann cells through the formation of radial lamellipodia, and, in MEFs, Rac1 SUMOylation is required for GTP loading/GTPase activity consequently leading to lamellipodia-ruffle formation. SUMOylation may control Schwann cell proliferation and differentiation by stimulating the autophosphorylation and subsequent activation of the Focal Adhesion Kinase (FAK). The stability and degradation of the effector of the Hippo pathway YAP could be modulated by SUMO conjugation in Schwann cells consequently affecting their functioning. Liver Kinase B1 (LKB1) is required for proper peripheral nerve myelination by Schwann cells, where SUMOylation may affect its interaction with other proteins (such as AMPK) and translocation to the nucleus. Similarly, SUMOylation of Lipin-1 enhances its nuclear shuttling and its function as a transcriptional co-activator in neurons; this might be a conserved mechanism in Schwann cells thus regulating lipid metabolism. SUMOylation could be also involved in the regulation of Schwann cell gene expression by affecting the transcriptional activity of *SOX10* and/or by epigenetic mechanisms mediated by the histone deacetylases HDAC1/2. *Krox20*, a critical regulator of Schwann cells, acts as a E3 ligase facilitating the SUMOylation of Nab proteins, possibly impacting on development and myelination of peripheral nerves. SUMOylation may also affect negative regulators of myelination, like the Notch Intracellular Domain (NICD); its modification by SUMO conjugation prevents shuttling to the nucleus and therefore reduces the transcription of downstream target genes. The downregulation of Notch signaling by *Krox20*, which notably can act as a SUMO ligase, is necessary for the normal onset of myelination. Although not directly demonstrated, the cell adhesion molecule L1 seems to be SUMOylated in Schwann cells, which promotes its cleavage by cathepsin E and subsequent translocation to the nucleus.

To date, there is no direct evidence in the literature that directly demonstrates a role for SUMOylation in Schwann cells. However, several proteins that are well known to be necessary for normal Schwann cell development and peripheral myelination have been found to be modified by SUMO conjugation in other contexts, as we outline below. Although our knowledge regarding the functional significance of SUMOylation in myelinating glia is very limited, these targets are promising candidates that may be similarly regulated by SUMO modification in Schwann cells, consequently impacting on crucial mechanisms of differentiation, radial sorting, myelin formation, and/or maintenance. In the first part of this article, we focus on substrate proteins that are important for Schwann cell development and myelination and that were found to be regulated by SUMO conjugation in other cell types (summarized in Figure 2). Subsequently, we review the few studies that suggest SUMOylation might regulate Schwann cell function. Finally, we speculate on the potential implications of dysregulation of this pathway in diseases affecting peripheral myelin.

## 2. Putative SUMOylable Substrates in Schwann Cells

### 2.1. Functional Consequences of SOX10 SUMOylation and Role in the Neural Crest

SOXE transcription factors (*SOX8*, *SOX9*, *SOX10*) are regulators of neural crest cell development [18]. In particular, *SOX10* is essential for specification of Schwann cells from the neural crest and is required at every stage of Schwann cell development [18,19]. Inactivating mutations of the *SOX10* gene in mice, which are associated with peripheral demyelinating neuropathy-central dysmyelinating leukodystrophy-Waardenburg syndrome-Hirschsprung disease (PCWH) in humans, prevent the generation of Schwann cells. The consequent lack of peripheral glial cells results in severe degeneration of sensory and motor neurons [20]. Interestingly, a yeast two-hybrid screen identified the conjugating enzyme *Ubc9* and *Sumo1* as SOXE interacting proteins [21]. *SOX10* has two SUMOylation consensus sites in *Xenopus*; indeed, in gastrula stages, *SOX10* is conjugated with *Sumo1* at K44 in the N-terminus as well as at K333 in the activation domain [21]. Finally, and most importantly, targeted mutation of these consensus sites, which blocks *SOX10* SUMOylation, affects the normal development of the neural crest in *Xenopus* embryos [21].

These findings were later extended to mammalian cells. *SOX10* is SUMOylated in HeLa cells in at least two of the three predicted consensus sites, but, in this case, *SOX10* was found to be indistinctly conjugated with either *Sumo1*, *Sumo2*, or *Sumo3* [22]. SUMOylation of *SOX10* seems to reduce the autonomous regulation of target gene transcription, such as *GJB1* (which encodes the gap junction protein connexin32 [Cx32], expressed by Schwann cells and associated with X-linked Charcot–Marie–Tooth disease), without preventing its shuttling to the nucleus or binding to regulatory regions of DNA. In support of this, targeted mutation of *SOX10* SUMOylation sites increased the expression of *GJB1* [22]. In addition, *SOX10* can also bind the *GJB1* promoter in a dimeric configuration with its cofactor *Krox20* (discussed below), consequently regulating gene expression [23]. Indeed, blocking *SOX10* SUMOylation increased the synergistic cooperation with *Krox20* upon *GJB1* promoter activation [22]. Similarly, abolishing SUMO conjugation upregulates expression of *MITF* (Microphthalmia-associated transcription factor) *MITF* by enhancing *SOX10* interaction with the cofactor paired box 3 (PAX3) [22]. This evidence indicates that SUMOylation of *SOX10* not only represses its autonomous transcriptional activity but also its synergistic transactivating activity upon partnering with *Krox20* and PAX3. 

Although SUMO-conjugation of *SOX10* in Schwann cells has not been directly reported, this could be a key SUMOylable candidate given its specific function in these glial cells. One cautionary note is that the systems utilized in the studies described above involve artificial overexpression that might not reflect physiological consequences of *SOX10* SUMOylation during normal development. That being said, SUMOylation seems to have a repressive effect on the cascade of *SOX10*-initiated events. Cx32 loss of function in mice causes a progressive demyelinating peripheral neuropathy [24], thus SUMOylation of *SOX10* might be detrimental for Schwann cell myelination. On the other hand, PAX3 has been shown to play a role in the path to differentiation into myelinating Schwann cells by repressing the expression of the myelin basic protein (*Mbp*) gene [25]. Moreover, PAX3 blocks the induction of *Krox20* by cyclic AMP and completely inhibits its ability to induce the expression of myelin genes in Schwann cells [26]. Therefore, if the regulatory mechanism of *SOX10*-PAX3 by SUMO-conjugation described in tumor cells is conserved in glia, it is also possible that SUMOylation may positively influence peripheral myelination. Nevertheless, a study in melanoma cells reported that, when *SOX10* is phosphorylated by ERK1/2, its SUMOylation at K55 is prevented, resulting instead in the inhibition of its transcriptional activity upon multiple target genes, including *MITF* [27]. This suggests that the repressive function of SUMOylation of *SOX10* might be cell-specific and may be dependent on the acceptor lysine residue, the number of lysine residues modified and on other PTMs. At a glance, it looks like the regulation of *SOX10* by SUMO protein conjugation might be an intricate mechanism to elucidate and the ultimate outcome of manipulations of this pathway may be difficult to predict.

### 2.2. SUMOylation Underlying the Krox20–NAB1/NAB2 Partnership

*Krox20* is another master transcription factor involved in the development of neural crest cells. In Schwann cells, *Krox20* is activated before the onset of myelination and its disruption stops differentiation at an early stage, hence preventing myelination in the PNS [28]. *Krox20* activation is required for the expression of myelin genes, like myelin protein zero (*MPZ*), and of genes involved in lipid biosynthesis [29,30,31,32]. Human *Krox20* gene mutations have been linked to peripheral myelin disorders like Charcot–Marie–Tooth (CMT) type 1D, Dejerine-Sottas syndrome (CMT3) and congenital hypomyelinating neuropathy [33]. This phenotype is recapitulated by genetically engineered mice carrying a hypomorphic *Krox20* allele causative of partial loss of function [34].

Several studies have shown that NGFI-A binding (Nab) proteins are important co-regulated interactors of *Krox20*. On one hand, *Krox20* positively regulates expression of Nab proteins in the hindbrain [35]. Consequently, genetic ablation of Nab1 or Nab2 causes a peripheral neuropathy similar to the one observed in *Krox20* knockout mice [36]. The *Krox20*-Nab complex is critical for peripheral myelin formation and, therefore, disruption of *Krox20*-Nab interaction by mutation of their interacting motifs hampers the transcription of critical genes necessary for proper peripheral myelination and causes a severe neuropathy [37,38]. At the same time, Nab proteins negatively control *Krox20* function in Schwann cells, thus downregulating the translation of several target genes in a regulatory feedback loop [39].

Surprisingly, *Krox20* interacts with *Ubc9* and then functions as a E3 ligase itself, facilitating the conjugation of Nab proteins with *Sumo1* in vitro [40]. This study also showed that SUMOylated Nab is recruited to promoters in the chromatin, repressing gene expression of *Krox20* target genes, whereas mutant non-SUMOylable Nab acts as a dominant negative protein increasing *Krox20*-driven expression. Thus, it seems that SUMOylation may add another control level to the complex co-regulation of *Krox20*/Nab function. Whether this is the case in Schwann cells is still to be determined. It is worth noting that the authors were not able to detect Nab SUMOylation with endogenous levels of *Sumo1* in P19 carcinoma cells, although this was observed after *Sumo1* overexpression. It is possible that SUMOylated Nab would be detectable under the right conditions and in the presence of the necessary signals and machinery, e.g., in developing sciatic nerves. Given the role of *Krox20*/Nab in the progression from promyelinating Schwann cells into myelination, we are tempted to speculate that failure of *Krox20*-mediated SUMOylation of Nab proteins might result in the arrest of Schwann cells in a proliferative stage and consequent peripheral neuropathy. This possibility needs to be investigated further.

### 2.3. Radial Sorting, Rac1, and SUMO

The actin cytoskeleton facilitates various essential biological functions in eukaryotic cells. Besides giving the cell shape and polarity, it also coordinates the mechanical forces required for cell movement and division. It is also known that members of the Rho family of guanosine small triphosphatases (GTPases) have regulatory roles in actin cytoskeleton rearrangement [41]. Particularly, the Rho GTPase Rac1 mediates cell polarization and lamellipodia formation in eukaryotic cells [42,43,44]. Reorganization of the actin cytoskeleton is a key process for PNS development, and Rac1 is essential for radial sorting and Schwann cell myelination [45]. During radial sorting, Schwann cells extend cytoplasmic protrusions towards axonal bundles in order to sort one individual axon, wrap it, and form myelin around it in a 1:1 relationship [46]. Crucial for this process, β1 integrin activates Rac1, which, in turn, results in PAK1 phosphorylation [47,48]. Rac1 Schwann cell conditional knockout (Rac1^cKO^) mice have a delay in radial sorting and severely impaired myelination [49]. Schwann cells isolated from Rac1^cKO^ mice, as well as rat Schwann cells treated with the Rac1 antagonist NSC23766, display an impairment in the extension of lamellipodia in vitro [49]. It has been postulated that Rac1 regulates the extension and stabilization of cytoplasmatic processes that mediate radial sorting and myelination, and defects in Rac1^cKO^ animals may be due to the inability of Schwann cells to form radial lamellipodia [49].

Interestingly, Rac1 can be modified by SUMOylation, and this PTM ultimately affects its function as well as cell behavior [50]. Particularly in immortalized cell lines, Rac1 interacts with the SUMO E3-ligase PIAS3 (Protein Inhibitor of Activated STAT3) promoting *Sumo1*-conjugation [50]. SUMO proteins can be covalently attached to the lysine residues K188, K183, and K184/186 within the C-terminal polybasic region (PBR) of Rac1. Replacement of the acceptor lysines by arginines (Rac1ΔSUMO), which abolishes most of the Rac1 SUMOylation in vitro, demonstrated that SUMOylation of Rac1 is required for its optimal GTP loading and activity. More importantly, blocking Rac1 SUMOylation has consequences on cell physiology. Rac1-null mouse embryonic fibroblasts (MEFs) are unable to form lamellipodia-membrane ruffles and are defective in cell migration. While transfection of Rac1-null MEFs with wildtype Rac1 almost completely rescues these defects, transfection of Rac1ΔSUMO (non-SUMOylable) constructs only rescues the failure of lamellipodia-ruffle formation partially [50].

Given the similarities in β1 integrin/Rac1/PAK1 axis activation in developing Schwann cells and in the study describing the association of Rac1 SUMOylation with lamellipodia formation, it is plausible that SUMO-mediated modulation of this signaling pathway could be conserved in the PNS. Interestingly, β1 integrin has a predicted SUMO interaction motif, which provides a theoretical link to SUMOylated Rac1. Although there is no direct evidence for the necessity of Rac1 SUMOylation to activate PAK1 in Schwann cells, the PBR of Rac1 is implicated in binding certain effectors, for example, PAK1 [51]. This speculation is strengthened by another study that found that *Sumo1* knockdown by siRNA impairs lamellipodia formation in fibroblast-like synoviocytes from patients with rheumatoid arthritis, concomitantly with reduced Rac1/PAK1 activation [52]. We propose that the role of Rac1 in the formation of radial lamellipodia in Schwann cells could be regulated by SUMOylation, an exciting hypothesis that warrants further investigation.

Alternatively, in tumor cells, another possible scenario has been described. In this case, a mutant p53 involved in tumorigenesis interacts with Rac1 preventing its interaction with SUMO-specific protease 1 (SENP1), which in turn inhibits de-SUMOylation therefore prolonging Rac1 activation [53]. Using a similar strategy as in [50] with wildtype and non-SUMOylable Rac1 constructs, these authors show that Rac1 SUMOylation is necessary for tumor cell migration and invasion [53]. Nevertheless, this mechanism might be more distant from Schwann cell physiology in non-pathological conditions. Hence, we are inclined to hypothesize that, in Schwann cells, Rac1 SUMOylation may be more likely involved in process extension and radial sorting, as proposed above.

### 2.4. SUMO-FAK and Schwann Cell Proliferation/Differentiation

The focal adhesion kinase (FAK) also forms an actin-associated complex with β1 integrin in Schwann cells [54]. Activation of FAK is initiated by autophosphorylation on the tyrosine residue Y397, which promotes the recruitment of other proteins and the activation of multiple signaling pathways. In vitro, tyrosine phosphorylation of FAK increases as Schwann cells form basal lamina and differentiate [54]. In vivo, myelination by Schwann cells lacking FAK is severely impaired due to insufficient cell proliferation and premature differentiation [55,56]. In a yeast two-hybrid screen, the N-terminal domain of FAK was found to interact with the protein inhibitor of activated STAT1 (PIAS1) [57]. In transfected cells, FAK is SUMOylated on K152, stimulating its autophosphorylation [57]. Therefore, it is plausible that SUMO modification may regulate FAK activation, thus promoting Schwann cell proliferation and preventing promiscuous differentiation.

### 2.5. Epigenetic Regulation by HDAC1/2 SUMOylation

Schwann cells are also controlled by epigenetic mechanisms [58]. In this context, histone deacetylases (HDACs) 1 and 2 play important roles in developmental myelination and myelin maintenance. Mice lacking both HDACs 1 and 2 (HDAC1/2) exhibit severe myelin deficiency with Schwann cell development arrested at the immature stage [59,60,61]. As a consequence of HDAC1/2 loss, transcription of target genes is affected. HDAC1/2 act cooperatively with NF-κB to regulate expression of many genes in Schwann cells, such as genes involved in differentiation and myelin encoding genes [59]. HDAC1/2 direct the specification of neural crest cells into peripheral glia by controlling the expression of PAX3 and, in turn, its target genes [61]. In adulthood, HDAC1/2 are also required for maintaining paranodal and nodal integrity, and their ablation causes peripheral nerve demyelination [62].

HDACs are regulated by many PTMs including SUMOylation [63]. Both HDAC1 and 2 were previously found to be SUMO-modified, e.g., [64,65,66,67,68]. SUMOylation of HDAC2 promotes NF-κB-dependent gene expression in a pathway that may help cancer cells escape cell cycle regulation [69]. Interestingly, in Schwann cells, NF-κB and HDAC1/2 act cooperatively to regulate the transcriptional programming of chromatin for myelination [59]. Thus, we hypothesize that SUMOylation may also be involved in this epigenetic regulatory mechanism of glia. It is worth noting that there is a certain degree of SUMO protein specificity for HDAC1. Conjugation of HDAC1 with *Sumo1*, but not *Sumo2*, promotes HDAC1 ubiquitination and subsequent degradation [70]. In non-tumorigenic mammary epithelial cells, HDAC1 is preferentially conjugated to *Sumo1* leading to HDAC1 proteolysis, whereas, in breast cancer cells, HDAC1 is predominantly conjugated to *Sumo2*, promoting HDAC1 protein stability; the specificity for SUMO proteins is dictated by the PIASγ ligase. The functions of HDAC1/2 in Schwann cells are also specific: HDAC2 activates the transcriptional program of myelination in synergy with *SOX10*, whereas HDAC1 controls Schwann cell survival by regulating the levels of active β-catenin [60]. These SUMO and HDAC specificities are to be taken into account when considering their possible interactions in Schwann cell differentiation and myelination. A final consideration is that HDAC2 seems to be able to interact with other SUMOylated proteins via SIMs, such as the transcription factor Elk1 and the translation initiation factor eIF4E (reviewed in [63]). In this way, SUMO proteins may also regulate transcription and translation through non-covalent interactions with HDAC, possibly contributing to Schwann cell function.

### 2.6. Taking Myelination down a Notch

Notch signaling is fundamental for glial development and myelination in both the CNS and PNS [71]. Notch receptors comprise four members, all of which are type I transmembrane proteins. Upon ligand binding, Notch receptors are cleaved intracellularly by secretases and the Notch intracellular domain (NICD) tranlocates into the nucleus to activate gene transcription [72]. In the PNS, Notch promotes the generation of Schwann cells from precursors and regulates the size of the immature Schwann cell pool by controlling proliferation [73]. NICD levels are downregulated at the start of myelination in sciatic nerves, likely causing Notch signaling inactivation (which hampers proliferation) in myelinating Schwann cells. Thus, Notch inhibits developmental myelination, and *Krox20*-dependent downregulation of Notch signaling is obligatory for the normal onset of myelination [73].

Interestingly, NICD1 is modified by SUMO in the nucleus of transfected HeLa cells, tethering it to this subcellular compartment but paradoxically attenuating its transcriptional activity [74]. At least one mechanism described for this transcriptional attenuation is through the interaction of SUMO-conjugated NICD1 with HDAC4. Similarly, in Schwann cells, HDAC1/2 can inhibit Notch signaling, thus regulating differentiation and myelination [75]. An analogous mechanism in Schwann cells is an attractive possibility: SUMOylation might simultaneously modulate the activity of opposing regulators like HDACs (discussed above) and NOTCH. This would have to be investigated. It is important to mention that another study showed that, if SUMO tag deconjugation from NICD1 by SENP1 is abolished, prolonged NOTCH1 signaling activation may become pathological over time [76]. This can be interpreted as SUMOylation having a pivotal regulatory action upon of NOTCH1; whether or not this is the case in Schwann cells remains to be elucidated.

### 2.7. Fat SUMO Wrestlers: Lipins

Lipid metabolism is important for myelinating glial cells as 70–85% of myelin composition is lipids [77]. Lipin-1 is a protein encoded by the *Lpin1* gene [78] with two isoforms produced by alternative mRNA splicing: Lipin-1α and Lipin-1β [79]. Lipin-1 functions as a Mg^2+^-dependent phosphatidic acid phosphohydrolase (PAP) enzyme, as well as a nuclear transcriptional coactivator [80]. It has been associated with the regulation of cellular lipid metabolism in many tissues including Schwann cells [81,82]. *Lpin1* mutant mice show a peripheral neuropathy characterized by demyelination, Schwann cell dedifferentiation and proliferation [83,84]. In neurons, Lipin-1α and Lipin-1β can be SUMOylated at two consensus motifs, and this modification was found to enrich Lipin-1 nuclear localization, where it acts as a transcriptional coactivator of the peroxisome proliferator activated receptor gamma coactivator 1α (PGC-1α) [85]. Interestingly, PGC-1α plays a role in Schwann cells, promoting mitochondrial stability during differentiation and myelination [86]. To date, the transcriptional function of Lipin-1 and its SUMOylation were further extended to brain tissue and, to a lesser extent, liver and muscle [85]. However, this still needs to be confirmed in Schwann cells since there are no reports of Lipin-1 SUMOylation in the PNS. Moreover, the downstream targets and implications in Schwann cells in particular would need to be identified. Nevertheless, it is interesting to find a lipid metabolism protein among the putative substrates for SUMO conjugation in myelinating glia.

### 2.8. The SUMO Wrestling Hippo

The Yes associated protein (YAP) is one effector of the Hippo pathway which integrates chemical and mechanical stimuli to regulate cell proliferation and apoptosis. When the Hippo pathway is activated, YAP is phosphorylated and sequestered in the cytoplasm, thus preventing interaction with the transcription factor TEAD and subsequently suppressing transcription of target genes [87]. The Hippo pathway plays a key role in PNS development and YAP conditional Schwann cell knockout mice present impairments in peripheral myelination [4].

Two studies report the SUMOylation of YAP [88,89]. In tumor cells, SUMOylation of YAP prevents its ubiquitination and subsequent degradation, which ultimately stabilizes the protein and extends its half-life [88]. This mechanism is believed to mediate cisplastin-induced cell death. Similarly, the YAP inhibitor verteporfin was shown to inhibit cell growth and induce cell death, but, contrarily to the previous paper, YAP SUMOylation was associated with its degradation in this case [89]. These findings are difficult to translate to Schwann cell development because YAP stabilization by SUMOylation might be just a consequence of chemotherapy-induced apoptosis of tumor cells. Whether SUMOylation affects the Hippo pathway in the PNS during development or in disease is still to be determined.

### 2.9. SUMOylation of LKB1 and Its Possible Role in Schwann Cell Metabolism

The liver kinase B1 (LKB1) is a major upstream kinase of the energy sensor AMP-activated protein kinase (AMPK). LKB1 is suggested to reprogram Schwann cells from a glycolytic to oxidative metabolism as they differentiate. This metabolic shift increases mitochondrial metabolism and lipogenesis, and may be necessary for normal myelination as evidenced by the hypomyelination of peripheral axons found in LKB1 knockout mice [90]. LKB1-mediated metabolic shift also impacts on the ability of Schwann cells to provide trophic support to axons [91]. LKB1 is asymmetrically localized to the Schwann cell-axon interface and co-localizes with the polarity protein Par-3. This localization is dependent on the phosphorylation of LKB1 at the serine residue S431 and is necessary for developmental myelination [92]. However, LKB1 can also be modified by SUMOylation in non-glial cells. LKB1 is conjugated to *Sumo2* at K178 hampering LKB1 nucleocytoplasmic shuttling in hepatocarcinoma cells, in turn promoting their survival [93]. Additionally, metabolic stress also triggers an increase in *Sumo1* conjugation to LKB1 at K178 in HEK293 cells [94]. *Sumo1* modification of LKB1 is essential for promoting its interaction with AMPK facilitated by a SIM; this process is necessary for AMPK activation. Mutation of the LKB1 SUMOylation site (K178R) causes defective AMPK signaling and mitochondrial function, inducing death in energy-deprived cells [94]. Taken together, this suggests that SUMOylation may possibly have a role in Schwann cell metabolism possibly through LKB1-AMPK signaling.

### 2.10. Crosstalk between SUMO and Kinases

To date, many other intracellular signaling pathways have been involved in Schwann cell myelination. Briefly, the main cascade transducers are: PI3K/Akt/mTOR, PLCγ, MAPK/ERK, and cAMP/PKA (see [95]). There are many examples in the literature of crosstalk between these pathways. Given the complexity of these interactions and the lack of direct evidence in Schwann cells, we will not discuss this topic thoroughly here. That being said, it is worth mentioning that SUMOylation might affect the intracellular cascade of events during myelination and vice versa. Just to cite some examples, the SUMO E3 ligases PIASxβ and PIAS3 interact with PLC-γ1 and cooperate to SUMOylate it in T-cells [96]. SUMO conjugation induces the activation of Akt [97] and SUMO-modified Akt regulates global SUMOylation and substrate specificity through phosphorylation of *Ubc9* and *Sumo1* in a positive feedback loop [98]. Conversely, knockdown of *Sumo1* in microglial cells results in decreased expression of PI3K and phosphorylation of AKT [99]. However, PIASxα functions as a SUMO E3 ligase for the PI3K negative regulator PTEN and its PTM blocks ubiquitination extending PTEN half-life [100].

MEK SUMOylation blocks ERK activation by disrupting the specific docking interaction between MEK and ERK [101], yet SUMOylation of upstream components of this pathway leads to the activation of MEK/MAPK [102]. As discussed above, ERK-mediated phosphorylation regulates *SOX10* SUMOylation in melanoma [27] and activation of the ERK MAP kinase pathway leads to Elk-1 deSUMOylation followed by loss of HDAC2-mediated repression [103]. Finally, the cAMP/PKA pathway has also been shown to positively and negatively impact on the SUMOylation of several substrates [104,105,106,107]. The interaction between SUMOylation and phosphorylation may be particularly intricate and much remains to be explored.

## 3. SUMO and Its Conjugation in Schwann Cells

### 3.1. L1 Cleavage and Possible SUMOylation in Schwann Cells

As mentioned at the beginning of this article, the literature on SUMOylation in Schwann cells is scarce. There are only two reports pointing to the potential involvement of this PTM in peripheral myelinating glia, the first of which is discussed next. The L1 cell adhesion molecule (L1CAM or simply L1) is a transmembrane protein, a member of the immunoglobulin superfamily. In Schwann cells, L1 promotes process formation, proliferation, and migration in vitro [108,109]. In vivo, the contribution of Schwann cell L1 to normal myelination is negligible compared to neuronal L1 (reviewed in [1]) and its role in regeneration after injury reported by different groups are somewhat contradictory and harder to reconcile [108,110]. Nevertheless, this is one of the two cases in the literature that suggests that SUMOylation may play a role in peripheral glia.

In cultured neurons, cathepsin E cleaves L1 generating a 30 kDa fragment (L1-30) that is imported into the nucleus [111]. In Schwann cells, protease inhibition, that presumably prevents the generation of L1-30, impairs migration, differentiation, and myelination in vitro. The authors also showed the presence of mono-*Sumo2/3* conjugated L1-30 in transfected HEK293 cells, and mutation of the putative acceptor residue (K1172) within a predicted consensus site also abolishes the generation of L1-30 and subsequent nuclear tethering. Taking these findings together, it can be hypothesized that, during myelination, SUMOylation may mediate L1 cleavage and subsequent translocation of L1-30 to the nucleus of Schwann cells, where it may modulate gene expression. We can even speculate that SUMOylation of L1 may facilitate its interaction with cathepsin E by non-covalent stabilization through SIMs, with subsequent proteolytic generation of L1-30. However, some considerations must be taken into account. First, it is important to mention that the SUMOylation of L1-30 was only shown in transfected HEK293 cells overexpressing the substrate and SUMO proteins. SUMO conjugation in physiological conditions would have strengthened the mechanism proposed in [111]. Second, since the abrogation of SUMO conjugation in the mutated K1172 L1-30 was not shown, it cannot be ruled out that another PTM may take place in said lysine residue. It is plausible that a different mechanism might contribute to the generation and nuclear translocation of L1-30. Finally, this study did not provide direct evidence of L1 SUMOylation in Schwann cells. This is particularly important because the existence of L1-30 was discovered in neurons, which express the full-length form of L1, whereas Schwann cells have a shorter, alternatively spliced version [112]. Thus, the modification of L1 by SUMO conjugation might be quite different depending on the cell type. In fact, evidence suggests that it is actually the neuronal L1 that is responsible for proper Schwann cell-axon adhesion, most likely through heterophilic interactions [113]. The cell-specific functions of L1 and its different forms in neurons and glia are important factors when considering the possible consequences of SUMOylation coming into play.

### 3.2. Sumo2 in Axo-Glial Interaction

As mentioned earlier, the processes of radial sorting and myelination require Schwann cells to interact with neuronal axons followed by subsequent induction of signaling pathways. The first empirical clue pointing to SUMOylation as a key process in peripheral myelination comes from a sophisticated in vitro model that identifies novel proteins involved in the early interaction between axons and Schwann cells [114,115]. Briefly, Schwann cells are cultured on a porous surface and stimulated to extend pseudopods towards a neuronal membrane preparation containing triggering axonal signals. The pseudopod subfraction can be removed and its content analyzed by proteomics. One of the proteins enriched in Schwann cells pseudopods is *Sumo2*, as well as ten other proteins known to bind or be directly regulated by *Sumo2* [114]. This finding suggests that SUMOylation may be involved in early axo-glial interaction and possibly in Schwann cell myelination. One of the first neighbors of *Sumo2* in the pseudopod interactome is the 70-kDa heat shock protein 8 (HSPA8) also known as 70-kDa heat shock cognate protein (HSC70) [114]. HSPA8, as a chaperone, facilitates the proper folding of newly translated and misfolded proteins, prevents protein aggregation, and degrades mutant or misfolded proteins. In this way, it is involved in important cellular processes like signal transduction, apoptosis, autophagy, protein homeostasis, and cell growth and differentiation [116,117]. HSPA8 is modified at K512 by covalent attachment of *Sumo1* or *Sumo2* [118,119]. Notably, HSPA8 has been implicated in myelination by oligodendrocytes, the myelinating glia of the central nervous system. Targeted knockdown with antisense oligonucleotides or pharmacological inhibition of HSC70 impairs the synthesis of myelin basic protein (MBP) in rat oligodendrocytes during differentiation [120,121]. Therefore, based on the available literature, HSC70 is a potential substrate for SUMOylation in Schwann cells. Finally, when the pool of proteins enriched in the pseudopod fraction was analyzed using the predictive algorithm GPS-SUMO, we found that ~70% of these proteins have putative SUMOylation sites (e.g., prohibitin2, a multifaceted protein that is necessary for axonal sorting and peripheral myelination [114]), whereas ~60% of these proteins are predicted to have SUMO interacting motifs (e.g., β1 integrin, an essential molecule in Schwann cells discussed above, see also [122]). Of note, these results were obtained using the most stringent predictive conditions to reduce the number of false positives. This suggests that there might be many targets modulated by SUMO modification. The identification of SUMOylated substrates in developing peripheral nerves is currently under investigation in our laboratory.

## 4. Disease

### 4.1. SUMOylation as a Convergent Mechanism in the Pathophysiology of Disease

**Figure 3:**
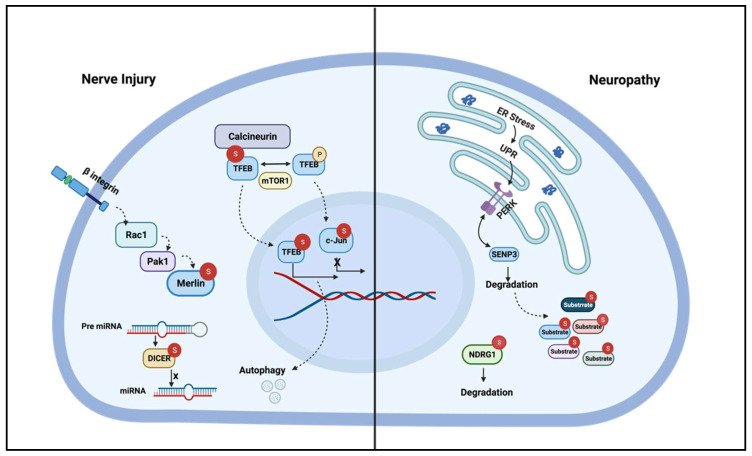
Putative SUMO substrate proteins in disease. (**Left**) SUMO-conjugated proteins are increased after peripheral nerve injury. Several proteins known to play roles in injury and regeneration have been reported to be SUMOylated in other contexts. For example, calcineurin promotes myelin autophagy via TFEB after injury, and the Calcineurin/NFAT signaling pathway becomes activated upon interaction with *Sumo2*. TFEB may also be regulated by SUMOylation. In this way, the increase in SUMO-conjugation after injury may stimulate nerve repair. c-JUN, which is essential for the reprogramming of Schwann cells during nerve regeneration. When SUMOylated, c-JUN stability is disturbed and its transcriptional activity is downregulated, this PTM also affects its interaction with other complex proteins, a plausible mechanism to restore Schwann cell homeostasis. The endoribonuclease DICER is SUMOylated hampering its miRNAs processing activity, but DICER is needed for Schwann cell transdifferentiation in response to nerve injury. SUMOylation of Merlin (downstream the beta1 Integrin/Rac1 signaling pathway shown in Figure 2) affects its subcellular localization and subsequently inhibits cell proliferation and migration, which could possibly control Schwann cell response to injury. (**Right**) In neuropathies such as Charcot–Marie–Tooth (CMT) 1A and 1B, the Unfolded Protein Response (UPR) pathway is activated. One hypothesis is that the UPR could lead to accumulation of SUMO conjugated proteins in CMT1 as PERK tags the deSUMOylating enzyme SENP3 for degradation. Finally, N-myc downstream regulated gene 1 (NDRG1) becomes unstable when conjugated to *Sumo2*, and mutations of this gene have been linked to CMT4D.

As mentioned above, the abundance of components of the SUMOylation machinery, as well as SUMOylated proteins during early brain development, suggests a role for this PTM in the CNS [14,123,124]. In addition, protein SUMOylation has been implicated in neurodegenerative disorders like Parkinson’s disease [125]. Furthermore, some of the putative substrates described above are also involved in neurological disorders. For instance, SUMOylation of HDAC1 is a natural mechanism to protect neurons against Amyloid-β toxicity in a mouse model of Alzheimer’s disease [126]. Therefore, it is also possible that alterations of SUMOylation may contribute to the pathophysiology of peripheral neuropathies (see Figure 3), and this pathway could become a therapeutic target in the future.

### 4.2. SUMOylation during Peripheral Nerve Injury and Regeneration

Contrary to the brain, the PNS has an extraordinary ability to regenerate thanks to the plasticity of Schwann cells and their transition between differentiated and dedifferentiated states, a process known as transdifferentiation. A recent study suggests a potential role of SUMOylation in nerve regeneration. After sciatic nerve injury, the levels of *Sumo1* and *Sumo2* in conjugated and free forms are upregulated in a time-dependent manner, concomitantly with increased *Ubc9* expression [127]. Although this report is mostly descriptive, there are several SUMOylable substrates in the literature that might be behind Schwann cell-mediated regeneration.

The transcription factor c-Jun is an essential activator of molecular repair reprogramming of Schwann cells during peripheral nerve injury and regeneration [128,129]. The activity of c-Jun is regulated by PTMs, including SUMOylation. SUMO attachment is mediated by two members of the PIAS family, PIAS1 and PIASxβ, which act as specific E3-like ligases by recruiting the *Ubc9* E2 enzyme to the respective substrate [130]. Strikingly, after c-Jun partners up with c-Fos, the AP-1 complex can still be SUMOylated at different sites, repressing its activity [131,132]. It is thus plausible that AP-1 SUMOylation could be a necessary regulatory mechanism to restore Schwann homeostasis and promote remyelination after injury.

Autophagy is a key step for nerve regeneration after injury by which Schwann cells clean the myelin debris [133]. We recently demonstrated that Calcineurin, which was originally thought to be important for PNS development, modulates autophagy by Schwann cells after sciatic nerve transection [134]. Interestingly, *Sumo2* interacts with Calcineurin without being covalently attached, tethers it to the nucleus, and activates Calcineurin-NFAT (for Nuclear Factor of Activated T-cells) signaling in neonatal rat cardiomyocytes [134]. It has been shown that Calcineurin regulates autophagy through the regulation of the transcription factor EB (TFEB) [135]. Interestingly, TFEB and TEF3 are also SUMOylated in vitro [136]. Taking evidence together, we hypothesize that increased levels of *Sumo2* after injury may activate this pathway to stimulate Schwann cell-mediated myelin autophagy and nerve repair. It is important to note that the injury study [127] did not distinguish between Schwann cell and axonal SUMOylation in the sciatic nerves. The increase in SUMO proteins could be attributed to either cell type, and even to infiltrating macrophages. The importance of cell autonomy and neurons is discussed below.

Micro RNAs (miRNAs) are important regulators of Schwann cell functions. A more distant candidate for SUMO modification that may also be involved in injury repair is the endoribonuclease DICER, the enzyme responsible for the biogenesis of miRNAs in the cell. DICER plays multiple roles in Schwan cells: it is necessary for radial sorting and developmental myelination, myelin maintenance in the adulthood, and regeneration after nerve injury [137,138,139]. It was shown that genetic ablation of DICER causes delays in Schwann cell cycling between differentiation states after peripheral nerve crush, therefore delaying remyelination [140]. At least in macrophages exposed to cigarette smoke, reduced DICER activity (and consequently reduced levels of miRNAs) seems to occur concomitantly with its SUMOylation [141]. Whereas the abundance of unmodified DICER decreases in macrophages from smokers compared to non-smokers, there is a shift towards the presence of higher molecular weight forms of this protein, probably representing PTMs of DICER. However, it is not clear if SUMOylation of DICER is a pathogenic mechanism or if it is intended to protect macrophages, but it fails to restore homeostasis. The authors even postulated that SUMO-modification of DICER might be related to autophagy, which is hampered by cigarette smoke, thus leading to impaired protein aggregate clearance [142]. Based on the body of evidence discussed thoroughly in this article, we speculate that the possibility of SUMOylation as a protective mechanism in peripheral nerve injury is more likely to be the case.

Another potential target of SUMO is the tumor suppressor Merlin/NF2, which in development acts downstream of Rac1 contributing to Schwann cell myelination [48]. Of note, Rac1 also plays a role in nerve injury [143]. Merlin localizes to the plasma membrane where it complexes with β1 integrin and Paxillin and helps organize the cytoskeleton of Schwann cells [144]. This anchorage to the plasma membrane circumvents its degradation [144]. Merlin also prevents Schwann cell proliferation [145,146]. Whereas Merlin/NF2 null animals display only a transient delay in developmental myelination, these animals have extensive failure of axonal regeneration and remyelination in the PNS, as well as aberrant and sustained Schwann cell proliferation [147]. These results were later extended using Schwann cell-specific mutant mice that express a dominant negative form of Merlin/NF2 and display delayed recovery after sciatic nerve crush [148]. Merlin can be SUMOylated on K76 in stably transfected cells and in tumor cells, immortalized lines, and MEFs [149]. The non-SUMOylable K76R mutant aggregates in the cytoplasm unlike wildtype Merlin which predominantly associates with the plasma membrane, suggesting that SUMOylation may determine Merlin’s subcellular localization. In addition, SUMO-conjugation of Merlin is necessary to suppress cell proliferation and migration. These observations together support the notion that SUMOylation could be a mechanism to control timely recovery after nerve injury, maybe mediated by Merlin/NF2 among other substrates.

On a final note, many proteins that are involved in nerve regeneration also play roles in developmental myelination. Other honorable mentions that were discussed thoroughly above are Rac1 [150], FAK [151], HDAC1/2 [152], NOTCH1 [153], YAP [154], and L1 [110].

### 4.3. Possible Implications of SUMO in Charcot–Marie–Tooth Disease

Peripheral neuropathies, which can be inherited or acquired, affect as much as 8% of the adult population and there is no curative treatment available [155]. Here, we focus on Charcot–Marie–Tooth (CMT) disease, which is the most commonly inherited neurological disorder, and is characterized by loss of nerve fibers and motor or sensory disability.

Among CMT subtypes, CMT1A is due to a chromosomal duplication of the peripheral myelin protein 22kD (PMP22) gene, whereas CMT1B is caused by dominant genetic mutations of myelin protein zero (P0). Findings in preclinical models have identified the unfolded protein response (UPR) as an important pathway in the pathogenesis of CMT1B. While initially the UPR plays a protective role by triggering mechanisms to return to homeostasis, if overwhelmed, it leads to cellular dysfunction. Mice carrying a S63del P0 mutation recapitulate the core symptomatology of CMT1B, namely demyelination, motor impairment, and reduced nerve conduction velocity [156]. S63del mice show an UPR in Schwann cells as revealed by high CHOP immunoreactivity, as well as XBP1, ATF6, and PERK activation. Most likely, the UPR is triggered by accumulation of P0 protein in the endoplasmic reticulum (ER). The UPR is pathogenic, as its modulation ameliorates the disease [157,158,159]. Additionally, the TremblerJ mutant, a model of CMT1A in which misfolded PMP22 protein is accumulated in the ER, also displays an upregulated UPR [160].

Interestingly, in cultured neurons, the UPR triggered by oxygen and glucose deprivation results in increased SUMOylation with *Sumo2/3* but not *Sumo1* [161]. UPR-activated PERK tags the de-SUMOylating enzyme SENP3 (which is specific for *Sumo2/3* chains) for degradation, thus causing the accumulation of *Sumo2/3*-conjugated proteins. This mechanism protects cells from apoptosis via accumulation of SUMOylated dynamin-related protein 1 (Drp-1) and suppression of cytochrome C release from the mitochondria [161]. To date, there is no reported evidence on SUMOylation abnormalities in CMT, but it is possible that *Sumo2/3*-conjugated proteins may be elevated by a similar mechanism. Yet another question arises: would SUMOylation be pathogenic or could it be a protective? In addition to the role of PERK/SENP3 in the UPR, ATF6 and XBP1 were found to be SUMOylated due to accumulation of misfolded proteins in the ER (another feature of CMT1), negatively regulating their translational activity [162,163]. Taken together, these findings further support a protective role of SUMOylation by acting as “turn-off” signal for the UPR when it is pathogenically active, as is the case of CMT1B [2,159]. Of note, SENP3 also negatively regulates autophagy triggered by starvation stress in the liver [164]; autophagy is another interesting pathway discussed above.

A more direct connection can be proposed between SUMOylation and CMT4D, a chromosomal recessive demyelinating polyneuropathy, resulting from mutations in the N-myc downstream-regulated gene 1 (NDRG1) [165]. NDRG1 is expressed at particularly high levels in Schwann cells, and, although its physiological functions are not completely understood, it is likely involved in myelin maintenance [166,167]. In cancer, NDRG1 suppresses metastasis without affecting primary tumorigenesis and, interestingly, is regulated by SUMOylation. NDRG1 is post-translationally modified preferentially by *Sumo2* at the major SUMO acceptor site K14, decreasing its stability [168]. If this mechanism is conserved in Schwann cells and is involved in the pathophysiology of CMT4D, then blocking SUMO conjugation and/or stimulation of SENP-mediated deSUMOylation may be beneficial for this disease. This hypothesis needs to be investigated.

## 5. Final Remarks

### 5.1. SUMOylation: The Good, the Bad, or the Ugly?

As was discussed throughout this perspective article, the consequences of protein modification by SUMO conjugation in Schwann cells are not entirely known. Moreover, several questions need to be addressed: is SUMOylation beneficial or detrimental in development? At least for radial sorting, it seems like SUMOylation may contribute to the formation of radial lamellipodia in Schwann cells through Rac1 modification [50]. This is supported by the observation of *Sumo2* enrichment in Schwann cells protrusions towards neuronal membranes, which can be attributed to free or conjugated *Sumo2* proteins attached to the substrates [114]. This PTM may also be necessary for Schwann cell metabolism and mitochondrial function via LKB1 [94] among other substrates. Therefore, blocking this PTM would potentially be deleterious for normal PNS development. On the other hand, SUMO-conjugation of transcription regulators such as *Krox20* and *Sox10* [22,40] might be necessary to switch off pro-myelinating cascades. Similarly, SUMOylation may promote Schwann cell proliferation and prevent premature differentiation by modification of FAK1 [57]. Hence, one could propose that sustained SUMOylation in the PNS beyond development may be pathogenic, although this is merely speculative.

A final question is whether *Sumo1* and *Sumo2/3* have similar or different functions in Schwann cells. For instance, the literature discussed above is mixed in terms of which SUMO protein (or combination thereof) is conjugated to a particular substrate. It is plausible that some proteins are conjugated with a specific SUMO protein or indistinctly conjugated to any of them. Moreover, SUMO proteins can form polymeric chains through the attachment of a SUMO molecule to an internal lysine moiety of another SUMO molecule, which can also be terminated by the addition of a *Sumo1* unit [169]. Nevertheless, some degree of specificity during the development of the PNS development is possible. In the rat brain, *Ubc9* expression spikes at embryonic day 15 (E15), and it is then gradually downregulated reaching basal levels around postnatal day 7 (P7). *Sumo1*-conjugated proteins are abundant in the embryonic brain but not in neonates, whereas *Sumo2/3* tagged proteins are highly enriched postnatally [14]. The consequences of this PTM on the substrate protein might be quite different depending on the nature of the SUMO tag. While the specificity of the SUMO protein to be attached to the substrate remains to be determined, it is proposed to be dictated by the target itself. In addition, research has not provided conclusive evidence yet on SUMO protein specificity for the substrates of interest in Schwann cells. The possibility of differential roles for *Sumo1* and *Sumo2/3* could help reconcile some of the contradictions discussed here. Indeed, the role of SUMOylation and SUMO proteins in Schwann cell development and developmental myelination remains to be elucidated.

### 5.2. Could SUMOylation Be a Therapeutic Target for Charcot–Marie–Tooth Disease?

In CMT1A, PMP22 aggregates associate with autophagosomes [170]. In addition, cultured fibroblasts from CMT1A patients exhibit an increase in the autophagy marker LC3-II and the lysosomal marker LAMP1 [171]. Modulation of autophagy can improve myelination in CMT1A models [172,173,174,175]. Moreover, increasing evidence connects SUMOylation and autophagy [176], for instance, the E1 enzyme inhibitor ginkgolic acid C15:1 removes aggresomes by promoting autophagy [177,178]. The Rho GTPase Rac1 is an exciting target for SUMOylation in disease. SUMOylation of Rac1 has been recently found to inhibit autophagy [178]. Thus, we hypothesize that preventing SUMOylation of Rac1 may stimulate autophagy, which could be a useful therapeutic approach.

However, other evidence suggests that excessive accumulation of SUMOylated proteins could initiate neurodegeneration [179]. SUMOylation has been associated with protein aggregation with implications in diseases like Alzheimer’s and Huntington’s [180]. Furthermore, it has been reported that SUMOylation can promote the formation of pathogenic aggresomes in amyotrophic lateral sclerosis (ALS) and Parkinson’s disease [181,182,183,184]. Moreover, the inhibition of SUMOylation reduces these cytoplasmic protein aggregates and is emerging as a potential therapeutic option [177,184]. Interestingly, cells isolated from CMT1A patients with elevated levels of PMP22 expression display intracellular protein aggregates as compared to cells from unaffected individuals [171]. In vitro, PMP22 mutants form spontaneous aggregates in Schwann cells [185]. The presence of aggresomes was also found in two mouse models of CMT1A, the TremblerJ, and the PMP22-C22 overexpressor [170,186,187,188]. These cytosolic PMP22 aggresome-like structures associate with the molecular chaperone HSPA8 and recruit lysosomes facilitating their elimination by an autophagy-mediated mechanism [187]. As discussed thoroughly above, HSPA8 is one of the enriched proteins in Schwann cell pseudopods and a putative SUMOylation substrate that in oligodendrocytes is needed for myelin formation [120,121]. Finally, knockdown of DICER (another SUMOylable target discussed above) results in increased levels of PMP22 [189]. If SUMOylation is truly needed for DICER degradation, as it has been suggested [141], SUMO-modification of DICER could be involved in the pathophysiology of CMT1A. In the case of CMT1B, aggregation of the P0 protein is less documented, but nevertheless exists [190,191]. Although there is evidence that these protein aggregates are ubiquitinated, another intriguing possibility is that they might be SUMOylated.

In either case, it seems plausible to target SUMOylation to ameliorate CMT symptoms. SUMOylation is emerging as a convergent dysregulated pathway in many human diseases, thus there is constant progress in the development of new compounds that modulate its enzymes [192,193,194]. While most inhibitors of SUMO conjugation and deconjugation have been assayed only in vitro, some of them have also been evaluated in preclinical models of diverse illnesses (Table 1). For instance, the SUMOylation inhibitor Ginkgolic acid C15:1 has shown promising therapeutic effects for myocardial infarction, arthritis, and cancer in laboratory animals [178,195,196,197]. Similarly, anacardic acid is structurally analogous to ginkgolic acid and blocks the formation of E1-SUMO intermediates reducing tumor growth in mice, and the specific *Ubc9* inhibitor 2-D08 also has antiproliferative properties against tumor xenografts [198]. In addition, both ginkgolic acid and 2-D08 may have beneficial effects on liver disease [199,200]. Next generation of SAE inhibitors with therapeutic action against malignancies include the small molecules ML-93/792 and the first-in-class inhibitor TAK-981 [201,202], the latter being under clinical trial (NCT03648372 and NCT04065555). Other compounds have not been tested in vivo yet but display good inhibitory activity in vitro [194].

Preclinical studies of SENPs inhibitors are less abundant and none of them have reached the clinical phase yet [205]. Some promising compounds to evaluate in mouse models of CMT are the natural SENP1 inhibitors Triptolide and Momordin Ic, which suppress prostate cancer cell proliferation in mice [203,204]. Although not tested in the context of disease, the SENP2 inhibitor Ebselen crosses the blood–brain barrier after acute injection increasing SUMOylation in the brain [206], thus it may be taken into consideration for the treatment of peripheral neuropathies since it likely crosses the blood–nerve barrier. However, first, we must understand the role of SUMOylation in the pathophysiology of CMT. If SUMOylation has a protective function during the UPR observed in CMT, inhibitors’ SENPs could have a beneficial effect on this neuropathy. Conversely, if SUMOylation of myelin proteins contributes to the formation of aggresomes, inhibitors of the enzymes E1 and E2 may alleviate the phenotype, possibly by the modulation of autophagy. In support of this, ginkgolic acid C15:1 has shown a potential therapeutic application in Parkinson’s disease since it promotes autophagy-dependent clearance of intracellular α-synuclein aggregates in vitro [177], and it can also induce autophagy in tumor-bearing mice [178]. Preclinical trials in CMT1A and 1B mouse models with either pharmacological inhibitors of SUMO conjugation or SENPs will shed light onto potential new therapeutic approaches.

### 5.3. A Cautionary Note about Neuronal SUMO

Some of the studies reviewed above, like [127], have not dissected the individual contribution of different cell types in the reported changes of SUMOylation. SUMO proteins play important roles in neurons. In the case of the PNS, SUMO conjugation is necessary for axonal transportation of mRNA in a dynein-mediated process, and abrogation of SUMOylation by means of mutation in acceptor lysine blocks retrograde transport of the RNA-binding protein La [207]. Neuronal SUMOylation is also important in peripheral neuropathies. For example, sensory neurons of diabetic patients and diabetic mice display changes in the SUMOylation status of metabolic enzymes and ion channels [208]. SUMOylation protects sensory neuron function as demonstrated by conditional ablation of the conjugating enzyme *Ubc9* in peripheral sensory neurons, causing accelerated neuropathology [208]. Thus, while in vivo analysis provides a holistic view of pathophysiology of nerve injury and regeneration, it does not reflect cell-autonomous effects, unless cell-specific conditional knockout models are used. In vitro studies are also useful to investigate cell-specific mechanisms and can provide insight into the role of SUMOylation in either Schwann cells or neurons [114]. However, these approaches may not recapitulate the complexity of the biological processes at play during the development of a multicellular organism as complex as a mammal. Schwann cell conditional knockout mouse models are robust tools for these types of analyses as well as for modeling disease.

### 5.4. Final Comment

Although the direct evidence suggesting a role of SUMOylation in Schwann cells is scarce, this is an exciting pathway to investigate. Based on the literature, SUMOylation may affect a plethora of targets in the PNS, from transcription factors to kinases. By doing so, it may regulate a variety of cellular processes, like gene expression, intracellular signal transduction, metabolism and lipid biosynthesis, cell–cell interaction, migration, and morphology. Ultimately, this may affect Schwann cell development, radial sorting, and myelination. Finally, SUMOylation may also be involved in the pathophysiology of peripheral neuropathies and regeneration after injury; therefore, this PTM might shed light on novel therapeutic approaches to human diseases by directly targeting components of its machinery or by the identification and modulation of newly discovered substrate proteins.

## Figures and Tables

**Table 1:** Inhibitors of SUMOylation enzymes with therapeutic properties in preclinical animal models.

Compound	Description	Target	Preclinical Therapeutic Action	References
Ginkgolic acid C15:1	Alkylphenol isolated from *Ginkgo biloba* leaves	E1 (SAE)	Alleviates cardiac fibrosis induced by myocardial infarction	[195]
			Attenuates severity of collagen-induced arthritis	[196]
			Inhibits growth of breast cancer xenografts	[196]
			Inhibits progression of oral squamous cell carcinoma	[197]
			Impedes induced liver fibrosis as co-adjuvant to other drugs	[199]
Anacardic acid	Phenolic lipid present in cashew nut shells	E1 (SAE)	Promotes differentiation of nonpromyelocytic acute myeloid leukemia	[198]
ML-93	Synthetic small molecule	E1 (SAE)	Reduces growth of pancreatic ductal adenocarcinoma	[201]
			Inhibits growth of diffuse large B-cell lymphoma and human colon cancer xenografts	[202]
ML-792	Synthetic small molecule	E1 (SAE)	Inhibits growth of diffuse large B-cell lymphoma and human colon cancer xenografts	[202]
TAK-981	Synthetic small molecule	E1 (SAE)	Inhibits growth of diffuse large B-cell lymphoma and human colon cancer xenografts	[202]
2-D08	Synthetic flavone	E2 (*Ubc9*)	Promotes differentiation of nonpromyelocytic acute myeloid leukemia	[198]
			Alleviates hepatic damage in transgenic mice	[200]
Triptolide	Diterpenoid epoxide from *Tripterygium wilfordii*	SENP1	Inhibits prostate cancer growth	[203]
Momordin Ic	Natural triterpenoid saponin	SENP1	Inhibits prostate cancer growth	[204]

## References

[1] Wilson E.R., Della-Flora Nunes G., Weaver M.R., Frick L.R., Feltri M.L. (2020). Schwann cell interactions during the development of the peripheral nervous system. Dev. Neurobiol..

[2] Musner N., Sidoli M., Zambroni D., Del Carro U., Ungaro D., D’Antonio M., Feltri M.L., Wrabetz L. (2016). Perk Ablation Ameliorates Myelination in S63del-Charcot–Marie–Tooth 1B Neuropathy. ASN Neuro.

[3] Ghidinelli M., Poitelon Y., Shin Y.K., Ameroso D., Williamson C., Ferri C., Pellegatta M., Espino K., Mogha A., Monk K. (2017). Laminin 211 inhibits protein kinase A in Schwann cells to modulate neuregulin 1 type III-driven myelination. PLoS Biol..

[4] Poitelon Y., Lopez-Anido C., Catignas K., Berti C., Palmisano M., Williamson C., Ameroso D., Abiko K., Hwang Y., Gregorieff A. (2016). YAP and TAZ control peripheral myelination and the expression of laminin receptors in Schwann cells. Nat. Neurosci..

[5] VerPlank J.J.S., Lokireddy S., Feltri M.L., Goldberg A.L., Wrabetz L. (2018). Impairment of protein degradation and proteasome function in hereditary neuropathies. Glia.

[6] Kim S., Maynard J.C., Sasaki Y., Strickland A., Sherman D.L., Brophy P.J., Burlingame A.L., Milbrandt J. (2016). Schwann Cell O-GlcNAc Glycosylation Is Required for Myelin Maintenance and Axon Integrity. J. Neurosci..

[7] Wang Y., Dasso M. (2009). SUMOylation and deSUMOylation at a glance. J. Cell Sci..

[8] Xu J., He Y., Qiang B., Yuan J., Peng X., Pan X.M. (2008). A novel method for high accuracy sumoylation site prediction from protein sequences. BMC Bioinform..

[9] Ijaz A. (2013). SUMOhunt: Combining Spatial Staging between Lysine and SUMO with Random Forests to Predict SUMOylation. Int. Sch. Res. Not..

[10] Nayak A., Muller S. (2014). SUMO-specific proteases/isopeptidases: SENPs and beyond. Genome Biol..

[11] Geiss-Friedlander R., Melchior F. (2007). Concepts in sumoylation: A decade on. Nat. Rev. Mol. Cell Biol..

[12] Ayaydin F., Dasso M. (2004). Distinct in vivo dynamics of vertebrate SUMO paralogues. Mol. Biol. Cell.

[13] Saitoh H., Hinchey J. (2000). Functional heterogeneity of small ubiquitin-related protein modifiers SUMO-1 versus SUMO-2/3. J. Biol. Chem..

[14] Loriol C., Parisot J., Poupon G., Gwizdek C., Martin S. (2012). Developmental regulation and spatiotemporal redistribution of the sumoylation machinery in the rat central nervous system. PLoS One.

[15] Nacerddine K., Lehembre F., Bhaumik M., Artus J., Cohen-Tannoudji M., Babinet C., Pandolfi P.P., Dejean A. (2005). The SUMO pathway is essential for nuclear integrity and chromosome segregation in mice. Dev. Cell.

[16] Zhang F.P., Mikkonen L., Toppari J., Palvimo J.J., Thesleff I., Janne O.A. (2008). Sumo-1 function is dispensable in normal mouse development. Mol. Cell Biol..

[17] Wang L., Wansleeben C., Zhao S., Miao P., Paschen W., Yang W. (2014). *Sumo2* is essential while *Sumo3* is dispensable for mouse embryonic development. EMBO Rep..

[18] Haldin C.E., LaBonne C. (2010). SoxE factors as multifunctional neural crest regulatory factors. Int. J. Biochem. Cell Biol..

[19] Srinivasan R., Sun G., Keles S., Jones E.A., Jang S.W., Krueger C., Moran J.J., Svaren J. (2012). Genome-wide analysis of EGR2/SOX10 binding in myelinating peripheral nerve. Nucleic Acids Res..

[20] Britsch S., Goerich D.E., Riethmacher D., Peirano R.I., Rossner M., Nave K.A., Birchmeier C., Wegner M. (2001). The transcription factor Sox10 is a key regulator of peripheral glial development. Genes Dev..

[21] Taylor K.M., Labonne C. (2005). SoxE factors function equivalently during neural crest and inner ear development and their activity is regulated by SUMOylation. Dev. Cell.

[22] Girard M., Goossens M. (2006). Sumoylation of the SOX10 transcription factor regulates its transcriptional activity. FEBS Lett..

[23] Bondurand N., Girard M., Pingault V., Lemort N., Dubourg O., Goossens M. (2001). Human Connexin 32, a gap junction protein altered in the X-linked form of Charcot-Marie-Tooth disease, is directly regulated by the transcription factor SOX10. Hum. Mol. Genet..

[24] Sargiannidou I., Vavlitou N., Aristodemou S., Hadjisavvas A., Kyriacou K., Scherer S.S., Kleopa K.A. (2009). Connexin32 mutations cause loss of function in Schwann cells and oligodendrocytes leading to PNS and CNS myelination defects. J. Neurosci..

[25] Kioussi C., Gross M.K., Gruss P. (1995). Pax3: A paired domain gene as a regulator in PNS myelination. Neuron.

[26] Doddrell R.D., Dun X.P., Moate R.M., Jessen K.R., Mirsky R., Parkinson D.B. (2012). Regulation of Schwann cell differentiation and proliferation by the Pax-3 transcription factor. Glia.

[27] Han S., Ren Y., He W., Liu H., Zhi Z., Zhu X., Yang T., Rong Y., Ma B., Purwin T.J. (2018). ERK-mediated phosphorylation regulates SOX10 sumoylation and targets expression in mutant BRAF melanoma. Nat. Commun..

[28] Topilko P., Schneider-Maunoury S., Levi G., Baron-Van Evercooren A., Chennoufi A.B., Seitanidou T., Babinet C., Charnay P. (1994). Krox-20 controls myelination in the peripheral nervous system. Nature.

[29] Jang S.W., LeBlanc S.E., Roopra A., Wrabetz L., Svaren J. (2006). In vivo detection of Egr2 binding to target genes during peripheral nerve myelination. J. Neurochem..

[30] Jang S.W., Srinivasan R., Jones E.A., Sun G., Keles S., Krueger C., Chang L.W., Nagarajan R., Svaren J. (2010). Locus-wide identification of Egr2/Krox20 regulatory targets in myelin genes. J. Neurochem..

[31] Leblanc S.E., Srinivasan R., Ferri C., Mager G.M., Gillian-Daniel A.L., Wrabetz L., Svaren J. (2005). Regulation of cholesterol/lipid biosynthetic genes by Egr2/Krox20 during peripheral nerve myelination. J. Neurochem..

[32] LeBlanc S.E., Jang S.W., Ward R.M., Wrabetz L., Svaren J. (2006). Direct regulation of myelin protein zero expression by the Egr2 transactivator. J. Biol. Chem..

[33] Warner L.E., Svaren J., Milbrandt J., Lupski J.R. (1999). Functional consequences of mutations in the early growth response 2 gene (EGR2) correlate with severity of human myelinopathies. Hum. Mol. Genet..

[34] Le N., Nagarajan R., Wang J.Y., Araki T., Schmidt R.E., Milbrandt J. (2005). Analysis of congenital hypomyelinating Egr2Lo/Lo nerves identifies Sox2 as an inhibitor of Schwann cell differentiation and myelination. Proc. Natl. Acad. Sci. USA.

[35] Mechta-Grigoriou F., Garel S., Charnay P. (2000). Nab proteins mediate a negative feedback loop controlling Krox-20 activity in the developing hindbrain. Development.

[36] Le N., Nagarajan R., Wang J.Y., Svaren J., LaPash C., Araki T., Schmidt R.E., Milbrandt J. (2005). Nab proteins are essential for peripheral nervous system myelination. Nat. Neurosci..

[37] Baloh R.H., Strickland A., Ryu E., Le N., Fahrner T., Yang M., Nagarajan R., Milbrandt J. (2009). Congenital hypomyelinating neuropathy with lethal conduction failure in mice carrying the Egr2 I268N mutation. J. Neurosci..

[38] Desmazieres A., Decker L., Vallat J.M., Charnay P., Gilardi-Hebenstreit P. (2008). Disruption of Krox20-Nab interaction in the mouse leads to peripheral neuropathy with biphasic evolution. J. Neurosci..

[39] Mager G.M., Ward R.M., Srinivasan R., Jang S.W., Wrabetz L., Svaren J. (2008). Active gene repression by the Egr2.NAB complex during peripheral nerve myelination. J. Biol. Chem..

[40] Garcia-Gutierrez P., Juarez-Vicente F., Gallardo-Chamizo F., Charnay P., Garcia-Dominguez M. (2011). The transcription factor Krox20 is an E3 ligase that sumoylates its Nab coregulators. EMBO Rep..

[41] Hall A. (1998). Rho GTPases and the actin cytoskeleton. Science.

[42] Eden S., Rohatgi R., Podtelejnikov A.V., Mann M., Kirschner M.W. (2002). Mechanism of regulation of WAVE1-induced actin nucleation by Rac1 and Nck. Nature.

[43] Westermarck J., Kahari V.M. (1999). Regulation of matrix metalloproteinase expression in tumor invasion. FASEB J..

[44] Bustelo X.R., Sauzeau V., Berenjeno I.M. (2007). GTP-binding proteins of the Rho/Rac family: Regulation, effectors and functions in vivo. Bioessays.

[45] Feltri M.L., Suter U., Relvas J.B. (2008). The function of RhoGTPases in axon ensheathment and myelination. Glia.

[46] Feltri M.L., Poitelon Y., Previtali S.C. (2016). How Schwann Cells Sort Axons: New Concepts. Neuroscientist.

[47] Sabra H., Brunner M., Mandati V., Wehrle-Haller B., Lallemand D., Ribba A.S., Chevalier G., Guardiola P., Block M.R., Bouvard D. (2017). Beta1 integrin-dependent Rac/group I PAK signaling mediates YAP activation of Yes-associated protein 1 (YAP1) via NF2/merlin. J. Biol. Chem..

[48] Guo L., Moon C., Niehaus K., Zheng Y., Ratner N. (2012). Rac1 controls Schwann cell myelination through cAMP and NF2/merlin. J. Neurosci..

[49] Nodari A., Zambroni D., Quattrini A., Court F.A., D’Urso A., Recchia A., Tybulewicz V.L., Wrabetz L., Feltri M.L. (2007). Beta1 integrin activates Rac1 in Schwann cells to generate radial lamellae during axonal sorting and myelination. J. Cell Biol..

[50] Castillo-Lluva S., Tatham M.H., Jones R.C., Jaffray E.G., Edmondson R.D., Hay R.T., Malliri A. (2010). SUMOylation of the GTPase Rac1 is required for optimal cell migration. Nat. Cell Biol..

[51] Lam B.D., Hordijk P.L. (2013). The Rac1 hypervariable region in targeting and signaling: A tail of many stories. Small GTPases.

[52] Lao M., Zhan Z., Li N., Xu S., Shi M., Zou Y., Huang M., Zeng S., Liang L., Xu H. (2019). Role of small ubiquitin-like modifier proteins-1 (SUMO-1) in regulating migration and invasion of fibroblast-like synoviocytes from patients with rheumatoid arthritis. Exp. Cell Res..

[53] Yue X., Zhang C., Zhao Y., Liu J., Lin A.W., Tan V.M., Drake J.M., Liu L., Boateng M.N., Li J. (2017). Gain-of-function mutant p53 activates small GTPase Rac1 through SUMOylation to promote tumor progression. Genes Dev..

[54] Chen L.M., Bailey D., Fernandez-Valle C. (2000). Association of beta 1 integrin with focal adhesion kinase and paxillin in differentiating Schwann cells. J. Neurosci..

[55] Grove M., Komiyama N.H., Nave K.A., Grant S.G., Sherman D.L., Brophy P.J. (2007). FAK is required for axonal sorting by Schwann cells. J. Cell Biol..

[56] Grove M., Brophy P.J. (2014). FAK is required for Schwann cell spreading on immature basal lamina to coordinate the radial sorting of peripheral axons with myelination. J. Neurosci..

[57] Kadare G., Toutant M., Formstecher E., Corvol J.C., Carnaud M., Boutterin M.C., Girault J.A. (2003). PIAS1-mediated sumoylation of focal adhesion kinase activates its autophosphorylation. J. Biol. Chem..

[58] Ma K.H., Svaren J. (2018). Epigenetic Control of Schwann Cells. Neuroscientist.

[59] Chen Y., Wang H., Yoon S.O., Xu X., Hottiger M.O., Svaren J., Nave K.A., Kim H.A., Olson E.N., Lu Q.R. (2011). HDAC-mediated deacetylation of NF-κB is critical for Schwann cell myelination. Nat. Neurosci..

[60] Jacob C., Christen C.N., Pereira J.A., Somandin C., Baggiolini A., Lotscher P., Ozcelik M., Tricaud N., Meijer D., Yamaguchi T. (2011). HDAC1 and HDAC2 control the transcriptional program of myelination and the survival of Schwann cells. Nat. Neurosci..

[61] Jacob C., Lotscher P., Engler S., Baggiolini A., Varum Tavares S., Brugger V., John N., Buchmann-Moller S., Snider P.L., Conway S.J. (2014). HDAC1 and HDAC2 control the specification of neural crest cells into peripheral glia. J. Neurosci..

[62] Brugger V., Engler S., Pereira J.A., Ruff S., Horn M., Welzl H., Munger E., Vaquie A., Sidiropoulos P.N., Egger B. (2015). HDAC1/2-Dependent P0 Expression Maintains Paranodal and Nodal Integrity Independently of Myelin Stability through Interactions with Neurofascins. Plos Biol..

[63] Segre C.V., Chiocca S. (2011). Regulating the regulators: The post-translational code of class I HDAC1 and HDAC2. J. Biomed. Biotechnol..

[64] Cheng J., Wang D., Wang Z., Yeh E.T. (2004). SENP1 enhances androgen receptor-dependent transcription through desumoylation of histone deacetylase 1. Mol. Cell Biol..

[65] Ouyang J., Gill G. (2009). SUMO engages multiple corepressors to regulate chromatin structure and transcription. Epigenetics.

[66] Park M.A., Seok Y.J., Jeong G., Lee J.S. (2008). *Sumo1* negatively regulates BRCA1-mediated transcription, via modulation of promoter occupancy. Nucleic Acids Res..

[67] Citro S., Chiocca S. (2016). Detection of Sumo Modification of Endogenous Histone Deacetylase 2 (HDAC2) in Mammalian Cells. Methods Mol. Biol..

[68] Joung H., Kwon S., Kim K.H., Lee Y.G., Shin S., Kwon D.H., Lee Y.U., Kook T., Choe N., Kim J.C. (2018). Sumoylation of histone deacetylase 1 regulates MyoD signaling during myogenesis. Exp. Mol. Med..

[69] Wagner T., Kiweler N., Wolff K., Knauer S.K., Brandl A., Hemmerich P., Dannenberg J.H., Heinzel T., Schneider G., Krämer O.H. (2015). Sumoylation of HDAC2 promotes NF-κB-dependent gene expression. Oncotarget.

[70] Citro S., Jaffray E., Hay R.T., Seiser C., Chiocca S. (2013). A role for paralog-specific sumoylation in histone deacetylase 1 stability. J. Mol. Cell Biol..

[71] Taveggia C., Feltri M.L., Wrabetz L. (2010). Signals to promote myelin formation and repair. Nat. Rev. Neurol..

[72] Kopan R., Ilagan M.X. (2009). The canonical Notch signaling pathway: Unfolding the activation mechanism. Cell.

[73] Woodhoo A., Alonso M.B., Droggiti A., Turmaine M., D’Antonio M., Parkinson D.B., Wilton D.K., Al-Shawi R., Simons P., Shen J. (2009). Notch controls embryonic Schwann cell differentiation, postnatal myelination and adult plasticity. Nat. Neurosci..

[74] Antila C.J.M., Rraklli V., Blomster H.A., Dahlstrom K.M., Salminen T.A., Holmberg J., Sistonen L., Sahlgren C. (2018). Sumoylation of Notch1 represses its target gene expression during cell stress. Cell Death Differ..

[75] Wu L.M., Wang J., Conidi A., Zhao C., Wang H., Ford Z., Zhang L., Zweier C., Ayee B.G., Maurel P. (2016). Zeb2 recruits HDAC-NuRD to inhibit Notch and controls Schwann cell differentiation and remyelination. Nat. Neurosci..

[76] Zhu X., Ding S., Qiu C., Shi Y., Song L., Wang Y., Wang Y., Li J., Wang Y., Sun Y. (2017). SUMOylation Negatively Regulates Angiogenesis by Targeting Endothelial NOTCH Signaling. Circ. Res..

[77] Poitelon Y., Kopec A.M., Belin S. (2020). Myelin Fat Facts: An Overview of Lipids and Fatty Acid Metabolism. Cells.

[78] Peterfy M., Phan J., Xu P., Reue K. (2001). Lipodystrophy in the fld mouse results from mutation of a new gene encoding a nuclear protein, lipin. Nat. Genet..

[79] Peterfy M., Phan J., Reue K. (2005). Alternatively spliced lipin isoforms exhibit distinct expression pattern, subcellular localization, and role in adipogenesis. J. Biol. Chem..

[80] Finck B.N., Gropler M.C., Chen Z., Leone T.C., Croce M.A., Harris T.E., Lawrence J.C., Kelly D.P. (2006). Lipin 1 is an inducible amplifier of the hepatic PGC-1alpha/PPARalpha regulatory pathway. Cell Metab..

[81] Verheijen M.H.G., Chrast R., Burrola P., Lemke G. (2003). Local regulation of fat metabolism in peripheral nerves. Genes Dev..

[82] Phan J., Reue K. (2005). Lipin, a lipodystrophy and obesity gene. Cell Metab..

[83] Nadra K., de Preux Charles A.S., Medard J.J., Hendriks W.T., Han G.S., Gres S., Carman G.M., Saulnier-Blache J.S., Verheijen M.H., Chrast R. (2008). Phosphatidic acid mediates demyelination in Lpin1 mutant mice. Genes Dev..

[84] Douglas D.S., Moran J.L., Bermingham J.R., Chen X.J., Brindley D.N., Soliven B., Beier D.R., Popko B. (2009). Concurrent Lpin1 and Nrcam mouse mutations result in severe peripheral neuropathy with transitory hindlimb paralysis. J. Neurosci..

[85] Liu G.H., Gerace L. (2009). Sumoylation regulates nuclear localization of lipin-1alpha in neuronal cells. PLoS One.

[86] Cowell R.M., Blake K.R., Inoue T., Russell J.W. (2008). Regulation of PGC-1alpha and PGC-1alpha-responsive genes with forskolin-induced Schwann cell differentiation. Neurosci. Lett..

[87] Feltri M.L., Weaver M.R., Belin S., Poitelon Y. (2021). The Hippo pathway: Horizons for innovative treatments of peripheral nerve diseases. J. Peripher. Nerv. Syst..

[88] Lapi E., Di Agostino S., Donzelli S., Gal H., Domany E., Rechavi G., Pandolfi P.P., Givol D., Strano S., Lu X. (2008). PML, YAP, and p73 are components of a proapoptotic autoregulatory feedback loop. Mol. Cell.

[89] Wang B., Shao W., Shi Y., Liao J., Chen X., Wang C. (2020). Verteporfin induced SUMOylation of YAP1 in endometrial cancer. Am. J. Cancer Res..

[90] Pooya S., Liu X., Kumar V.B., Anderson J., Imai F., Zhang W., Ciraolo G., Ratner N., Setchell K.D., Yoshida Y. (2014). The tumour suppressor LKB1 regulates myelination through mitochondrial metabolism. Nat. Commun..

[91] Beirowski B., Babetto E., Golden J.P., Chen Y.J., Yang K., Gross R.W., Patti G.J., Milbrandt J. (2014). Metabolic regulator LKB1 is crucial for Schwann cell-mediated axon maintenance. Nat. Neurosci..

[92] Shen Y.A., Chen Y., Dao D.Q., Mayoral S.R., Wu L., Meijer D., Ullian E.M., Chan J.R., Lu Q.R. (2014). Phosphorylation of LKB1/Par-4 establishes Schwann cell polarity to initiate and control myelin extent. Nat. Commun..

[93] Zubiete-Franco I., Garcia-Rodriguez J.L., Lopitz-Otsoa F., Serrano-Macia M., Simon J., Fernandez-Tussy P., Barbier-Torres L., Fernandez-Ramos D., Gutierrez-de-Juan V., Lopez de Davalillo S. (2019). SUMOylation regulates LKB1 localization and its oncogenic activity in liver cancer. EBioMedicine.

[94] Ritho J., Arold S.T., Yeh E.T. (2015). A Critical *Sumo1* Modification of LKB1 Regulates AMPK Activity during Energy Stress. Cell Rep..

[95] Salzer J.L. (2015). Schwann cell myelination. Cold Spring Harb Perspect. Biol..

[96] Wang Q.L., Liang J.Q., Gong B.N., Xie J.J., Yi Y.T., Lan X., Li Y. (2019). T Cell Receptor (TCR)-Induced PLC-γ1 Sumoylation via PIASxβ and PIAS3 SUMO E3 Ligases Regulates the Microcluster Assembly and Physiological Function of PLC-γ1. Front. Immunol..

[97] de la Cruz-Herrera C.F., Campagna M., Lang V., del Carmen Gonzalez-Santamaria J., Marcos-Villar L., Rodriguez M.S., Vidal A., Collado M., Rivas C. (2015). SUMOylation regulates AKT1 activity. Oncogene.

[98] Lin C.H., Liu S.Y., Lee E.H. (2016). SUMO modification of Akt regulates global SUMOylation and substrate SUMOylation specificity through Akt phosphorylation of Ubc9 and *Sumo1*. Oncogene.

[99] Saw G., Krishna K., Gupta N., Soong T.W., Mallilankaraman K., Sajikumar S., Dheen S.T. (2020). Epigenetic regulation of microglial phosphatidylinositol 3-kinase pathway involved in long-term potentiation and synaptic plasticity in rats. Glia.

[100] Wang W., Chen Y., Wang S., Hu N., Cao Z., Wang W., Tong T., Zhang X. (2014). PIASxα ligase enhances *Sumo1* modification of PTEN protein as a SUMO E3 ligase. J. Biol. Chem..

[101] Kubota Y., O’Grady P., Saito H., Takekawa M. (2011). Oncogenic Ras abrogates MEK SUMOylation that suppresses the ERK pathway and cell transformation. Nat. Cell Biol..

[102] Qu Y., Chen Q., Lai X., Zhu C., Chen C., Zhao X., Deng R., Xu M., Yuan H., Wang Y. (2014). SUMOylation of Grb2 enhances the ERK activity by increasing its binding with Sos1. Mol. Cancer.

[103] Yang S.H., Sharrocks A.D. (2004). SUMO promotes HDAC-mediated transcriptional repression. Mol. Cell.

[104] Li X., Vadrevu S., Dunlop A., Day J., Advant N., Troeger J., Klussmann E., Jaffrey E., Hay R.T., Adams D.R. (2010). Selective SUMO modification of cAMP-specific phosphodiesterase-4D5 (PDE4D5) regulates the functional consequences of phosphorylation by PKA and ERK. Biochem. J..

[105] Wang H., Zhang J., You G. (2019). Activation of Protein Kinase A Stimulates SUMOylation, Expression, and Transport Activity of Organic Anion Transporter 3. AAPS J..

[106] Cox B., Briscoe J., Ulloa F. (2010). SUMOylation by Pias1 regulates the activity of the Hedgehog dependent Gli transcription factors. PLoS One.

[107] Su Y.F., Shyu Y.C., Shen C.K., Hwang J. (2012). Phosphorylation-dependent SUMOylation of the transcription factor NF-E2. PLoS ONE.

[108] Lavdas A.A., Efrose R., Douris V., Gaitanou M., Papastefanaki F., Swevers L., Thomaidou D., Iatrou K., Matsas R. (2010). Soluble forms of the cell adhesion molecule L1 produced by insect and baculovirus-transduced mammalian cells enhance Schwann cell motility. J. Neurochem..

[109] Schulz F., Lutz D., Rusche N., Bastús N.G., Stieben M., Höltig M., Grüner F., Weller H., Schachner M., Vossmeyer T. (2013). Gold nanoparticles functionalized with a fragment of the neural cell adhesion molecule L1 stimulate L1-mediated functions. Nanoscale.

[110] Guseva D., Angelov D.N., Irintchev A., Schachner M. (2009). Ablation of adhesion molecule L1 in mice favours Schwann cell proliferation and functional recovery after peripheral nerve injury. Brain.

[111] Lutz D., Wolters-Eisfeld G., Schachner M., Kleene R. (2014). Cathepsin E generates a sumoylated intracellular fragment of the cell adhesion molecule L1 to promote neuronal and Schwann cell migration as well as myelination. J. Neurochem..

[112] Reid R.A., Hemperly J.J. (1992). Variants of human L1 cell adhesion molecule arise through alternate splicing of RNA. J. Mol. Neurosci..

[113] Haney C.A., Sahenk Z., Li C., Lemmon V.P., Roder J., Trapp B.D. (1999). Heterophilic binding of L1 on unmyelinated sensory axons mediates Schwann cell adhesion and is required for axonal survival. J. Cell Biol..

[114] Poitelon Y., Bogni S., Matafora V., Della-Flora Nunes G., Hurley E., Ghidinelli M., Katzenellenbogen B.S., Taveggia C., Silvestri N., Bachi A. (2015). Spatial mapping of juxtacrine axo-glial interactions identifies novel molecules in peripheral myelination. Nat. Commun..

[115] Poitelon Y., Feltri M.L. (2018). The Pseudopod System for Axon-Glia Interactions: Stimulation and Isolation of Schwann Cell Protrusions that Form in Response to Axonal Membranes. Methods Mol. Biol.

[116] Stricher F., Macri C., Ruff M., Muller S. (2013). HSPA8/HSC70 chaperone protein: Structure, function, and chemical targeting. Autophagy.

[117] Liu T., Daniels C.K., Cao S. (2012). Comprehensive review on the HSC70 functions, interactions with related molecules and involvement in clinical diseases and therapeutic potential. Pharm..

[118] Impens F., Radoshevich L., Cossart P., Ribet D. (2014). Mapping of SUMO sites and analysis of SUMOylation changes induced by external stimuli. Proc. Natl. Acad. Sci. USA.

[119] Hendriks I.A., D’Souza R.C., Yang B., Verlaan-de Vries M., Mann M., Vertegaal A.C. (2014). Uncovering global SUMOylation signaling networks in a site-specific manner. Nat. Struct. Mol. Biol..

[120] Aquino D.A., Lopez C., Farooq M. (1996). Antisense oligonucleotide to the 70-kDa heat shock cognate protein inhibits synthesis of myelin basic protein. Neurochem. Res..

[121] Aquino D.A., Peng D., Lopez C., Farooq M. (1998). The constitutive heat shock protein-70 is required for optimal expression of myelin basic protein during differentiation of oligodendrocytes. Neurochem. Res..

[122] Feltri M.L., Graus Porta D., Previtali S.C., Nodari A., Migliavacca B., Cassetti A., Littlewood-Evans A., Reichardt L.F., Messing A., Quattrini A. (2002). Conditional disruption of beta 1 integrin in Schwann cells impedes interactions with axons. J. Cell Biol..

[123] Watanabe M., Takahashi K., Tomizawa K., Mizusawa H., Takahashi H. (2008). Developmental regulation of Ubc9 in the rat nervous system. Acta Biochim. Pol..

[124] Hasegawa Y., Yoshida D., Nakamura Y., Sakakibara S. (2014). Spatiotemporal distribution of SUMOylation components during mouse brain development. J. Comp. Neurol..

[125] Anderson D.B., Zanella C.A., Henley J.M., Cimarosti H. (2017). Sumoylation: Implications for Neurodegenerative Diseases. Adv. Exp. Med. Biol..

[126] Tao C.C., Hsu W.L., Ma Y.L., Cheng S.J., Lee E.H. (2017). Epigenetic regulation of HDAC1 SUMOylation as an endogenous neuroprotection against Aβ toxicity in a mouse model of Alzheimer’s disease. Cell Death Differ..

[127] Zhang D.Y., Yu K., Yang Z., Liu X.Z., Ma X.F., Li Y.X. (2019). Variation in expression of small ubiquitin-like modifiers in injured sciatic nerve of mice. Neural Regen. Res..

[128] Arthur-Farraj P.J., Latouche M., Wilton D.K., Quintes S., Chabrol E., Banerjee A., Woodhoo A., Jenkins B., Rahman M., Turmaine M. (2012). c-Jun reprograms Schwann cells of injured nerves to generate a repair cell essential for regeneration. Neuron.

[129] Parkinson D.B., Bhaskaran A., Arthur-Farraj P., Noon L.A., Woodhoo A., Lloyd A.C., Feltri M.L., Wrabetz L., Behrens A., Mirsky R. (2008). c-Jun is a negative regulator of myelination. J. Cell Biol..

[130] Schmidt D., Muller S. (2002). Members of the PIAS family act as SUMO ligases for c-Jun and p53 and repress p53 activity. Proc. Natl. Acad. Sci. USA.

[131] Bossis G., Malnou C.E., Farras R., Andermarcher E., Hipskind R., Rodriguez M., Schmidt D., Muller S., Jariel-Encontre I., Piechaczyk M. (2005). Down-regulation of c-Fos/c-Jun AP-1 dimer activity by sumoylation. Mol. Cell Biol..

[132] Tempe D., Vives E., Brockly F., Brooks H., De Rossi S., Piechaczyk M., Bossis G. (2014). SUMOylation of the inducible (c-Fos:c-Jun)/AP-1 transcription complex occurs on target promoters to limit transcriptional activation. Oncogene.

[133] Gomez-Sanchez J.A., Carty L., Iruarrizaga-Lejarreta M., Palomo-Irigoyen M., Varela-Rey M., Griffith M., Hantke J., Macias-Camara N., Azkargorta M., Aurrekoetxea I. (2015). Schwann cell autophagy, myelinophagy, initiates myelin clearance from injured nerves. J. Cell Biol..

[134] Kao S.C., Wu H., Xie J., Chang C.P., Ranish J.A., Graef I.A., Crabtree G.R. (2009). Calcineurin/NFAT signaling is required for neuregulin-regulated Schwann cell differentiation. Science.

[135] Medina D.L., Di Paola S., Peluso I., Armani A., De Stefani D., Venditti R., Montefusco S., Scotto-Rosato A., Prezioso C., Forrester A. (2015). Lysosomal calcium signalling regulates autophagy through calcineurin and TFEB. Nat. Cell Biol..

[136] Miller A.J., Levy C., Davis I.J., Razin E., Fisher D.E. (2005). Sumoylation of MITF and its related family members TFE3 and TFEB. J. Biol. Chem..

[137] Gokbuget D., Pereira J.A., Opitz L., Christe D., Kessler T., Marchais A., Suter U. (2018). The miRNA biogenesis pathway prevents inappropriate expression of injury response genes in developing and adult Schwann cells. Glia.

[138] Pereira J.A., Baumann R., Norrmen C., Somandin C., Miehe M., Jacob C., Luhmann T., Hall-Bozic H., Mantei N., Meijer D. (2010). Dicer in Schwann cells is required for myelination and axonal integrity. J. Neurosci..

[139] Li T., Wang J., Wang H., Yang Y., Wang S., Huang N., Wang F., Gao X., Niu J., Li Z. (2018). The deletion of dicer in mature myelinating glial cells causes progressive axonal degeneration but not overt demyelination in adult mice. Glia.

[140] Viader A., Chang L.W., Fahrner T., Nagarajan R., Milbrandt J. (2011). MicroRNAs modulate Schwann cell response to nerve injury by reinforcing transcriptional silencing of dedifferentiation-related genes. J. Neurosci..

[141] Gross T.J., Powers L.S., Boudreau R.L., Brink B., Reisetter A., Goel K., Gerke A.K., Hassan I.H., Monick M.M. (2014). A microRNA processing defect in smokers’ macrophages is linked to SUMOylation of the endonuclease DICER. J. Biol. Chem..

[142] Monick M.M., Powers L.S., Walters K., Lovan N., Zhang M., Gerke A., Hansdottir S., Hunninghake G.W. (2010). Identification of an autophagy defect in smokers’ alveolar macrophages. J. Immunol..

[143] Park H.T., Feltri M.L. (2011). Rac1 GTPase controls myelination and demyelination. Bioarchitecture.

[144] Manetti M.E., Geden S., Bott M., Sparrow N., Lambert S., Fernandez-Valle C. (2012). Stability of the tumor suppressor merlin depends on its ability to bind paxillin LD3 and associate with β1 integrin and actin at the plasma membrane. Biol. Open.

[145] Denisenko N., Cifuentes-Diaz C., Irinopoulou T., Carnaud M., Benoit E., Niwa-Kawakita M., Chareyre F., Giovannini M., Girault J.A., Goutebroze L. (2008). Tumor suppressor schwannomin/merlin is critical for the organization of Schwann cell contacts in peripheral nerves. J. Neurosci..

[146] Lallemand D., Manent J., Couvelard A., Watilliaux A., Siena M., Chareyre F., Lampin A., Niwa-Kawakita M., Kalamarides M., Giovannini M. (2009). Merlin regulates transmembrane receptor accumulation and signaling at the plasma membrane in primary mouse Schwann cells and in human schwannomas. Oncogene.

[147] Mindos T., Dun X.P., North K., Doddrell R.D., Schulz A., Edwards P., Russell J., Gray B., Roberts S.L., Shivane A. (2017). Merlin controls the repair capacity of Schwann cells after injury by regulating Hippo/YAP activity. J. Cell Biol..

[148] Truong K., Ahmad I., Jason Clark J., Seline A., Bertroche T., Mostaert B., Van Daele D.J., Hansen M.R. (2018). Nf2 Mutation in Schwann Cells Delays Functional Neural Recovery Following Injury. Neuroscience.

[149] Qi Q., Liu X., Brat D.J., Ye K. (2014). Merlin sumoylation is required for its tumor suppressor activity. Oncogene.

[150] Jung J., Cai W., Lee H.K., Pellegatta M., Shin Y.K., Jang S.Y., Suh D.J., Wrabetz L., Feltri M.L., Park H.T. (2011). Actin polymerization is essential for myelin sheath fragmentation during Wallerian degeneration. J. Neurosci..

[151] Chang H.M., Shyu M.K., Tseng G.F., Liu C.H., Chang H.S., Lan C.T., Hsu W.M., Liao W.C. (2013). Neuregulin facilitates nerve regeneration by speeding Schwann cell migration via ErbB2/3-dependent FAK pathway. PLoS One.

[152] Brugger V., Duman M., Bochud M., Munger E., Heller M., Ruff S., Jacob C. (2017). Delaying histone deacetylase response to injury accelerates conversion into repair Schwann cells and nerve regeneration. Nat. Commun..

[153] El Bejjani R., Hammarlund M. (2012). Notch signaling inhibits axon regeneration. Neuron.

[154] Jeanette H., Marziali L.N., Bhatia U., Hellman A., Herron J., Kopec A.M., Feltri M.L., Poitelon Y., Belin S. (2021). YAP and TAZ regulate Schwann cell proliferation and differentiation during peripheral nerve regeneration. Glia.

[155] Martyn C.N., Hughes R.A. (1997). Epidemiology of peripheral neuropathy. J. Neurol. Neurosurg. Psychiatry.

[156] Wrabetz L., D’Antonio M., Pennuto M., Dati G., Tinelli E., Fratta P., Previtali S., Imperiale D., Zielasek J., Toyka K. (2006). Different intracellular pathomechanisms produce diverse Myelin Protein Zero neuropathies in transgenic mice. J. Neurosci..

[157] Pennuto M., Tinelli E., Malaguti M., Del Carro U., D’Antonio M., Ron D., Quattrini A., Feltri M.L., Wrabetz L. (2008). Ablation of the UPR-mediator CHOP restores motor function and reduces demyelination in Charcot-Marie-Tooth 1B mice. Neuron.

[158] D’Antonio M., Musner N., Scapin C., Ungaro D., Del Carro U., Ron D., Feltri M.L., Wrabetz L. (2013). Resetting translational homeostasis restores myelination in Charcot-Marie-Tooth disease type 1B mice. J. Exp. Med..

[159] Sidoli M., Musner N., Silvestri N., Ungaro D., D’Antonio M., Cavener D.R., Feltri M.L., Wrabetz L. (2016). Ablation of Perk in Schwann Cells Improves Myelination in the S63del Charcot-Marie-Tooth 1B Mouse. J. Neurosci..

[160] Okamoto Y., Pehlivan D., Wiszniewski W., Beck C.R., Snipes G.J., Lupski J.R., Khajavi M. (2013). Curcumin facilitates a transitory cellular stress response in Trembler-J mice. Hum. Mol. Genet..

[161] Guo C., Hildick K.L., Luo J., Dearden L., Wilkinson K.A., Henley J.M. (2013). SENP3-mediated deSUMOylation of dynamin-related protein 1 promotes cell death following ischaemia. EMBO J..

[162] Hou X., Yang Z., Zhang K., Fang D., Sun F. (2017). SUMOylation represses the transcriptional activity of the Unfolded Protein Response transducer ATF6. Biochem. Biophys Res. Commun..

[163] Chen H., Qi L. (2010). SUMO modification regulates the transcriptional activity of XBP1. Biochem. J..

[164] Liu K., Guo C., Lao Y., Yang J., Chen F., Zhao Y., Yang Y., Yang J., Yi J. (2020). A fine-tuning mechanism underlying self-control for autophagy: deSUMOylation of BECN1 by SENP3. Autophagy.

[165] Ricard E., Mathis S., Magdelaine C., Delisle M.B., Magy L., Funalot B., Vallat J.M. (2013). CMT4D (NDRG1 mutation): Genotype-phenotype correlations. J. Peripher. Nerv. Syst..

[166] King R.H., Chandler D., Lopaticki S., Huang D., Blake J., Muddle J.R., Kilpatrick T., Nourallah M., Miyata T., Okuda T. (2011). Ndrg1 in development and maintenance of the myelin sheath. Neurobiol. Dis..

[167] Okuda T., Higashi Y., Kokame K., Tanaka C., Kondoh H., Miyata T. (2004). Ndrg1-deficient mice exhibit a progressive demyelinating disorder of peripheral nerves. Mol. Cell Biol..

[168] Lee J.E., Kim J.H. (2015). SUMO modification regulates the protein stability of NDRG1. Biochem. Biophys. Res. Commun..

[169] Matic I., van Hagen M., Schimmel J., Macek B., Ogg S.C., Tatham M.H., Hay R.T., Lamond A.I., Mann M., Vertegaal A.C.O. (2008). In vivo identification of human small ubiquitin-like modifier polymerization sites by high accuracy mass spectrometry and an in vitro to in vivo strategy. Mol. Cell Proteom..

[170] Fortun J., Go J.C., Li J., Amici S.A., Dunn W.A., Notterpek L. (2006). Alterations in degradative pathways and protein aggregation in a neuropathy model based on PMP22 overexpression. Neurobiol. Dis..

[171] Lee S., Bazick H., Chittoor-Vinod V., Al Salihi M.O., Xia G., Notterpek L. (2018). Elevated Peripheral Myelin Protein 22, Reduced Mitotic Potential, and Proteasome Impairment in Dermal Fibroblasts from Charcot-Marie-Tooth Disease Type 1A Patients. Am. J. Pathol..

[172] Fortun J., Verrier J.D., Go J.C., Madorsky I., Dunn W.A., Notterpek L. (2007). The formation of peripheral myelin protein 22 aggregates is hindered by the enhancement of autophagy and expression of cytoplasmic chaperones. Neurobiol. Dis..

[173] Madorsky I., Opalach K., Waber A., Verrier J.D., Solmo C., Foster T., Dunn W.A., Notterpek L. (2009). Intermittent fasting alleviates the neuropathic phenotype in a mouse model of Charcot-Marie-Tooth disease. Neurobiol. Dis..

[174] Rangaraju S., Verrier J.D., Madorsky I., Nicks J., Dunn W.A., Notterpek L. (2010). Rapamycin activates autophagy and improves myelination in explant cultures from neuropathic mice. J. Neurosci..

[175] Nicks J., Lee S., Harris A., Falk D.J., Todd A.G., Arredondo K., Dunn W.A., Notterpek L. (2014). Rapamycin improves peripheral nerve myelination while it fails to benefit neuromuscular performance in neuropathic mice. Neurobiol. Dis..

[176] Vijayakumaran S., Pountney D.L. (2018). SUMOylation, aging and autophagy in neurodegeneration. Neurotoxicology.

[177] Vijayakumaran S., Nakamura Y., Henley J.M., Pountney D.L. (2019). Ginkgolic acid promotes autophagy-dependent clearance of intracellular alpha-synuclein aggregates. Mol. Cell Neurosci..

[178] Lorente M., Garcia-Casas A., Salvador N., Martinez-Lopez A., Gabicagogeascoa E., Velasco G., Lopez-Palomar L., Castillo-Lluva S. (2019). Inhibiting *Sumo1*-mediated SUMOylation induces autophagy-mediated cancer cell death and reduces tumour cell invasion via RAC1. J. Cell Sci..

[179] Lee L., Sakurai M., Matsuzaki S., Arancio O., Fraser P. (2013). SUMO and Alzheimer’s disease. Neuromolecular Med..

[180] Liebelt F., Vertegaal A.C. (2016). Ubiquitin-dependent and independent roles of SUMO in proteostasis. Am. J. Physiol. Cell Physiol..

[181] Fei E., Jia N., Yan M., Ying Z., Sun Q., Wang H., Zhang T., Ma X., Ding H., Yao X. (2006). SUMO-1 modification increases human SOD1 stability and aggregation. Biochem. Biophys. Res. Commun..

[182] Dangoumau A., Marouillat S., Burlaud Gaillard J., Uzbekov R., Veyrat-Durebex C., Blasco H., Arnoult C., Corcia P., Andres C.R., Vourc’h P. (2016). Inhibition of Pathogenic Mutant SOD1 Aggregation in Cultured Motor Neuronal Cells by Prevention of Its SUMOylation on Lysine 75. Neurodegener Dis..

[183] Oh Y., Kim Y.M., Mouradian M.M., Chung K.C. (2011). Human Polycomb protein 2 promotes α-synuclein aggregate formation through covalent SUMOylation. Brain Res..

[184] Maurel C., Chami A.A., Thépault R.A., Marouillat S., Blasco H., Corcia P., Andres C.R., Vourc’h P. (2020). A role for SUMOylation in the Formation and Cellular Localization of TDP-43 Aggregates in Amyotrophic Lateral Sclerosis. Mol. Neurobiol..

[185] Johnson J.S., Roux K.J., Fletcher B.S., Fortun J., Notterpek L. (2005). Molecular alterations resulting from frameshift mutations in peripheral myelin protein 22: Implications for neuropathy severity. J. Neurosci. Res..

[186] Ryan M.C., Shooter E.M., Notterpek L. (2002). Aggresome formation in neuropathy models based on peripheral myelin protein 22 mutations. Neurobiol. Dis..

[187] Fortun J., Dunn W.A., Joy S., Li J., Notterpek L. (2003). Emerging role for autophagy in the removal of aggresomes in Schwann cells. J. Neurosci..

[188] Fortun J., Li J., Go J., Fenstermaker A., Fletcher B.S., Notterpek L. (2005). Impaired proteasome activity and accumulation of ubiquitinated substrates in a hereditary neuropathy model. J. Neurochem..

[189] Verrier J.D., Lau P., Hudson L., Murashov A.K., Renne R., Notterpek L. (2009). Peripheral myelin protein 22 is regulated post-transcriptionally by miRNA-29a. Glia.

[190] Li J., Bai Y., Ianakova E., Grandis M., Uchwat F., Trostinskaia A., Krajewski K.M., Garbern J., Kupsky W.J., Shy M.E. (2006). Major myelin protein gene (P0) mutation causes a novel form of axonal degeneration. J. Comp. Neurol..

[191] Lee S.M., Olzmann J.A., Chin L.S., Li L. (2011). Mutations associated with Charcot-Marie-Tooth disease cause SIMPLE protein mislocalization and degradation by the proteasome and aggresome-autophagy pathways. J. Cell Sci..

[192] Yang Y., Xia Z., Wang X., Zhao X., Sheng Z., Ye Y., He G., Zhou L., Zhu H., Xu N. (2018). Small-Molecule Inhibitors Targeting Protein SUMOylation as Novel Anticancer Compounds. Mol. Pharm..

[193] Zhou Y., Ji C., Cao M., Guo M., Huang W., Ni W., Meng L., Yang H., Wei J.F. (2018). Inhibitors targeting the SUMOylation pathway: A patent review 2012–2015 (Review). Int. J. Mol. Med..

[194] Kroonen J.S., Vertegaal A.C.O. (2021). Targeting SUMO Signaling to Wrestle Cancer. Trends Cancer.

[195] Qiu F., Dong C., Liu Y., Shao X., Huang D., Han Y., Wang B., Liu Y., Huo R., Paulo P. (2018). Pharmacological inhibition of SUMO-1 with ginkgolic acid alleviates cardiac fibrosis induced by myocardial infarction in mice. Toxicol. Appl. Pharm..

[196] Wang C., Xiao Y., Lao M., Wang J., Xu S., Li R., Xu X., Kuang Y., Shi M., Zou Y. (2020). Increased SUMO-activating enzyme SAE1/UBA2 promotes glycolysis and pathogenic behavior of rheumatoid fibroblast-like synoviocytes. JCI Insight.

[197] Liu K., Wang X., Li D., Xu D., Li D., Lv Z., Zhao D., Chu W.F., Wang X.F. (2020). Ginkgolic Acid, a SUMO-1 Inhibitor, Inhibits the Progression of Oral Squamous Cell Carcinoma by Alleviating SUMOylation of SMAD4. Mol. Oncolytics.

[198] Baik H., Boulanger M., Hosseini M., Kowalczyk J., Zaghdoudi S., Salem T., Sarry J.E., Hicheri Y., Cartron G., Piechaczyk M. (2018). Targeting the SUMO Pathway Primes All-trans Retinoic Acid-Induced Differentiation of Nonpromyelocytic Acute Myeloid Leukemias. Cancer Res..

[199] Zhou J., Cui S., He Q., Guo Y., Pan X., Zhang P., Huang N., Ge C., Wang G., Gonzalez F.J. (2020). SUMOylation inhibitors synergize with FXR agonists in combating liver fibrosis. Nat. Commun..

[200] Huang J., Xie P., Dong Y., An W. (2021). Inhibition of Drp1 SUMOylation by ALR protects the liver from ischemia-reperfusion injury. Cell Death Differ..

[201] Biederstädt A., Hassan Z., Schneeweis C., Schick M., Schneider L., Muckenhuber A., Hong Y., Siegers G., Nilsson L., Wirth M. (2020). SUMO pathway inhibition targets an aggressive pancreatic cancer subtype. Gut.

[202] Langston S.P., Grossman S., England D., Afroze R., Bence N., Bowman D., Bump N., Chau R., Chuang B.C., Claiborne C. (2021). Discovery of TAK-981, a First-in-Class Inhibitor of SUMO-Activating Enzyme for the Treatment of Cancer. J. Med. Chem..

[203] Huang W., He T., Chai C., Yang Y., Zheng Y., Zhou P., Qiao X., Zhang B., Liu Z., Wang J. (2012). Triptolide inhibits the proliferation of prostate cancer cells and down-regulates SUMO-specific protease 1 expression. PLoS One.

[204] Wu J., Lei H., Zhang J., Chen X., Tang C., Wang W., Xu H., Xiao W., Gu W., Wu Y. (2016). Momordin Ic, a new natural SENP1 inhibitor, inhibits prostate cancer cell proliferation. Oncotarget.

[205] Tokarz P., Woźniak K. (2021). SENP Proteases as Potential Targets for Cancer Therapy. Cancers.

[206] Bernstock J.D., Ye D., Smith J.A., Lee Y.J., Gessler F.A., Yasgar A., Kouznetsova J., Jadhav A., Wang Z., Pluchino S. (2018). Quantitative high-throughput screening identifies cytoprotective molecules that enhance SUMO conjugation via the inhibition of SUMO-specific protease (SENP)2. FASEB J..

[207] van Niekerk E.A., Willis D.E., Chang J.H., Reumann K., Heise T., Twiss J.L. (2007). Sumoylation in axons triggers retrograde transport of the RNA-binding protein La. Proc. Natl. Acad. Sci. USA.

[208] Agarwal N., Taberner F.J., Rangel Rojas D., Moroni M., Omberbasic D., Njoo C., Andrieux A., Gupta P., Bali K.K., Herpel E. (2020). SUMOylation of Enzymes and Ion Channels in Sensory Neurons Protects against Metabolic Dysfunction, Neuropathy, and Sensory Loss in Diabetes. Neuron.

